# Bistability in a Metabolic Network Underpins the De Novo Evolution of Colony Switching in *Pseudomonas fluorescens*

**DOI:** 10.1371/journal.pbio.1002109

**Published:** 2015-03-12

**Authors:** Jenna Gallie, Eric Libby, Frederic Bertels, Philippe Remigi, Christian B. Jendresen, Gayle C. Ferguson, Nicolas Desprat, Marieke F. Buffing, Uwe Sauer, Hubertus J. E. Beaumont, Jan Martinussen, Mogens Kilstrup, Paul B. Rainey

**Affiliations:** 1 New Zealand Institute for Advanced Study, Massey University, Auckland, New Zealand; 2 Department of Environmental Microbiology, Eawag, Dübendorf, Switzerland; 3 Department of Environmental Systems Science, ETH Zürich, Zürich, Switzerland; 4 Santa Fe Institute, Santa Fe, New Mexico, United States of America; 5 Institute of Integrative Biology, ETH Zürich, Zürich, Switzerland; 6 Department of Systems Biology, Technical University of Denmark, Lyngby, Denmark; 7 Novo Nordisk Foundation Center for Biosustainability, Technical University of Denmark, Hørsholm, Denmark; 8 Laboratoire de Physique Statistique (UMR8550), Ecole Normale Superieure, Paris, France; 9 Institut de Biologie de l'ENS (IBENS UMR 8197), Ecole Normale Superieure, Paris, France; 10 Universite Paris Diderot, Paris, France; 11 Institute of Molecular Systems Biology, ETH Zürich, Zürich, Switzerland; 12 Department of Bionanoscience, Kavli Institute of Nanoscience, Delft University of Technology, Delft, The Netherlands; 13 Max-Planck Institute for Evolutionary Biology, Plön, Germany; HHMI, Massachusetts Institute of Technology, UNITED STATES

## Abstract

Phenotype switching is commonly observed in nature. This prevalence has allowed the elucidation of a number of underlying molecular mechanisms. However, little is known about how phenotypic switches arise and function in their early evolutionary stages. The first opportunity to provide empirical insight was delivered by an experiment in which populations of the bacterium *Pseudomonas fluorescens* SBW25 evolved, de novo, the ability to switch between two colony phenotypes. Here we unravel the molecular mechanism behind colony switching, revealing how a single nucleotide change in a gene enmeshed in central metabolism (*carB*) generates such a striking phenotype. We show that colony switching is underpinned by ON/OFF expression of capsules consisting of a colanic acid-like polymer. We use molecular genetics, biochemical analyses, and experimental evolution to establish that capsule switching results from perturbation of the pyrimidine biosynthetic pathway. Of central importance is a bifurcation point at which uracil triphosphate is partitioned towards either nucleotide metabolism or polymer production. This bifurcation marks a cell-fate decision point whereby cells with relatively high pyrimidine levels favour nucleotide metabolism (capsule OFF), while cells with lower pyrimidine levels divert resources towards polymer biosynthesis (capsule ON). This decision point is present and functional in the wild-type strain. Finally, we present a simple mathematical model demonstrating that the molecular components of the decision point are capable of producing switching. Despite its simple mutational cause, the connection between genotype and phenotype is complex and multidimensional, offering a rare glimpse of how noise in regulatory networks can provide opportunity for evolution.

## Abstract 2

The molecular basis of an experimentally evolved colony-level phenotype switch is revealed to affect a metabolic bifurcation point where nucleotides can be used for either cell division or capsule polymer synthesis.

## Author Summary

Phenotype switching—the ability to switch rapidly between phenotypic states—is an evolutionary survival strategy commonly used by organisms in the face of unpredictable environmental conditions. However, little is known about how phenotype switches emerge and function in their early evolutionary stages. A previous study observed the evolutionary emergence of colony morphology switching in *Pseudomonas fluorescens* populations in response to fluctuating selection. Here we describe the underlying molecular basis of this colony switching, providing the first account of the mechanism behind a real-time evolved phenotype switch. We show that colony switching in this instance is underpinned at the cellular level by high frequency ON/OFF expression of colanic acid-like capsules in response to varying levels of a metabolite. Biochemical assays revealed that capsule switching results from mutations that reduce concentrations of intermediates in a central metabolic pathway—the pyrimidine biosynthetic pathway. Of key importance is the partitioning of these metabolic resources between polymer production (leading to capsulation) and cell division (leading to noncapsulation); this bifurcation marks a decision point whereby cells with low metabolite levels divert resources towards polymer production, increasing the likelihood of switching to the capsulated state. As a greater proportion of cells become capsulated, colony switching emerges. These findings show that, while colony switching evolved with relative ease, the underlying molecular mechanism is surprisingly complex.

## Introduction

Life is constantly challenged by environmental change. Survival requires that organisms match their phenotype to current conditions. In predictable environments, this occurs by phenotypic acclimation [1]. In unpredictable environments, where the quality of environmental information is unreliable, stochastic phenotype switching allows organisms to hedge their evolutionary bets [2]. Bet hedging spreads the risk of being maladapted in the current environment among variable offspring, each of which has a chance of being fit in some future environment [3–5].

Bet-hedging strategies are widespread in nature, with examples from humans through to microbes [6–10]. Such strategies appear to be common in bacterial pathogens and commensals; when confronted with the challenge of survival in the face of an unpredictable host immune response, these bacteria have evolved mutational mechanisms that allow stochastic modulation of phenotype (contingency loci [11]). Typically, these involve short repetitive DNA sequences that affect expression of genes involved in critical interactions with the host. By virtue of their repeated nucleotide motifs, contingency loci are prone to polymerase slippage, resulting in localized hypermutation.

The sophistication of contingency loci begs questions as to their evolutionary origins. Given that pathogens and commensals are often derived from environmental reservoirs, it is likely that the ancestral state was a gene, or gene network, that was subject to environmental regulation. A recent experiment in which a plant saprophyte subject to fluctuating environmental conditions evolved, de novo, the capacity for stochastic phenotype switching [12] offers opportunities to understand how such switches originate. In this previous study, an experimental population of *Pseudomonas fluorescens* SBW25 was propagated under fluctuating conditions that mimicked essential aspects of the host immune response [13]. After multiple environmental reversals in which evolving populations experienced repeated bottlenecks and phenotypic exclusion, genotypes able to stochastically switch between different colony states were identified in two of 12 replicate lines. This work focuses on the first of these lineages.

**Fig 1:**
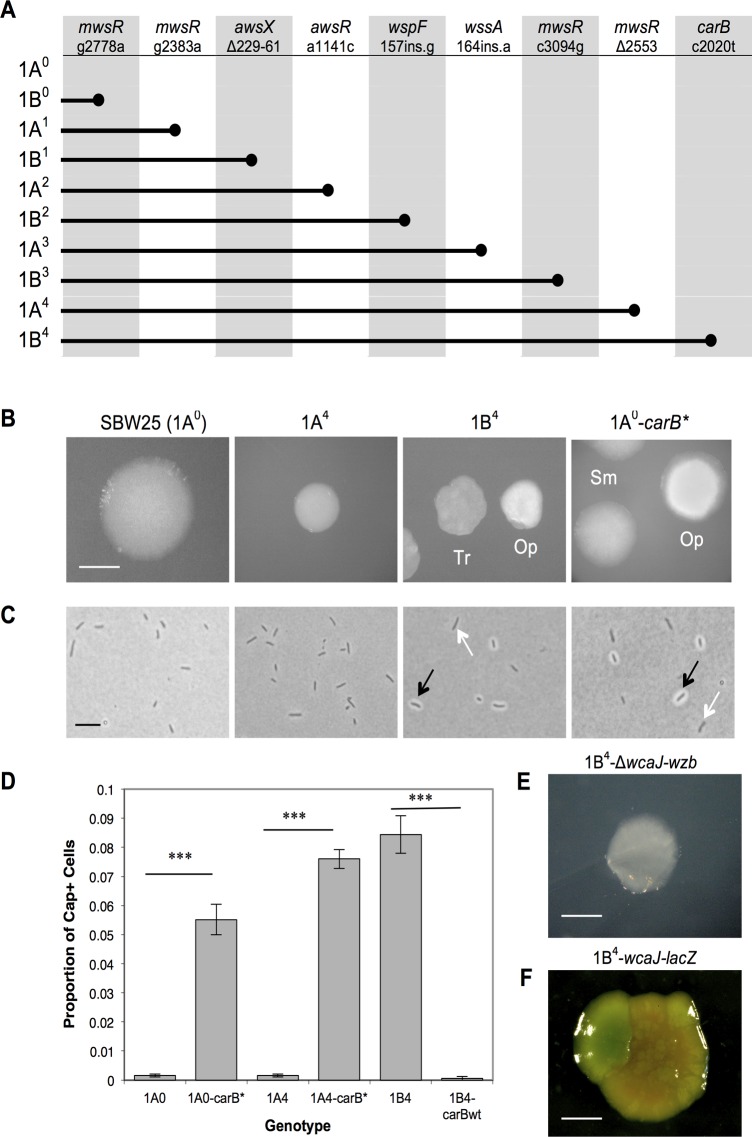
The evolutionary history, genotype, and phenotype of 1B^4^. (**A**) The evolutionary lineage from 1A^0^ (SBW25) to 1B^4^ consists of nine genotypes (rows). Each consecutive genotype contains an additional mutation (columns: *gene* and nucleotide change). Grey bars indicate the mutations present in each genotype; grey circles mark the first occurrence. (**B**) Colonies grown on King’s Medium B (KB) agar for 48 h. 1B^4^ and 1A^0^-carB* give dimorphic colonies: opaque (Op) and translucent (Tr; 1B^4^) or smooth (Sm; 1A^0^-carB*). Bar ~0.5 cm. Saturation and exposure of images was altered. (**C**) Light microscope images of cells counterstained with India ink. 1B^4^ and 1A^0^-carB* have Cap^-^ and Cap^+^ cells (white and black arrows). Bar ~4 μm. Saturation and exposure of images was altered. (**D**) Proportion of Cap^+^ cells in evolutionary series and engineered genotypes. Introduction of the c2020t *carB* mutation to ancestors increases capsulation (1A^0^-carB*, 1A^4^-carB*). Removal of the mutation from 1B^4^ (1B^4^-*carB*wt) reduces capsulation. Bars are means of five replicates, and error bars denote +/- 1 SE. Two sample *t* tests for the indicated pairs of genotypes revealed significant differences in capsulation levels (*p* < 0.001). (**E**) A 1B^4^-Δ*wcaJ-wzb* colony grown on KB medium. Bar ~0.5 cm. (**F**) A 1B^4^-*wcaJ-lacZ* colony grown on medium containing X-gal. Light microscopy was used to check blue sectors contained a greater proportion of Cap^+^ cells than elsewhere. Bar ~1 cm. Image contrast and exposure was altered.

The capacity to stochastically switch evolved after nine mutations (Fig. 1A). The first eight facilitated bouts of adaptation to different states of the fluctuating environment. The ninth, a single nucleotide change in *carB* (c2020t; encoding a subunit of CarAB, EC 6.3.5.5), gave rise to a genotype referred to as 1B^4^ (see Fig. 1A for details of evolutionary series genotype names) that switched at high frequency between two colony types (translucent and opaque; Fig. 1B). Further investigation showed that differences in colony type were attributable to the varying proportion of cells with different capsulation states (Cap^-^ and Cap^+^, respectively; Fig. 1C). In addition, allelic replacement studies showed the *carB* mutation alone was sufficient to generate stochastic colony and capsule switching in the wild-type background (Fig. 1D).

**Fig 2:**
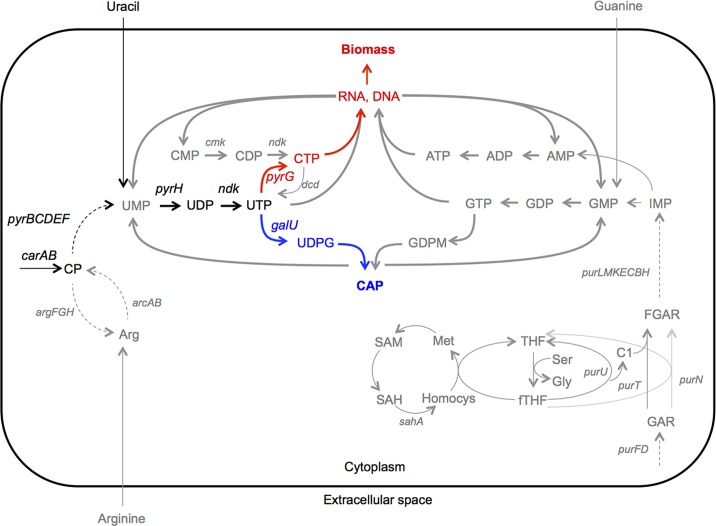
Intracellular metabolic pathways downstream of *carB*. The pyrimidine biosynthetic pathway is a molecular link between the mutant *carB* genotype and the CAP capsule phenotype. The switch-causing *carB* mutation reduces concentrations of intermediates in the pyrimidine biosynthesis pathway (shown in black), exposing a decision point at which uridine triphosphate (UTP) is used either by PyrG for nucleotide biosynthesis (leading to the Cap^-^ phenotype, components in red), or by GalU for polymer biosynthesis (generating Cap^+^ phenotype, components in blue). Metabolic pathways related to UTP biosynthesis are shown in grey. High-flux reactions recycling nucleotides are shown by thick arrows, and nucleotide biosynthetic pathways are shown by thin arrows. Figure based on amino acid homology with established *Escherichia coli* pathways [14].

Here we describe empirical and theoretical efforts to understand how a single mutation in a gene deeply enmeshed in central metabolism generates such a striking phenotype. The switch-causing mutation reduces concentrations of intermediates in the pyrimidine biosynthetic pathway, thus exposing a pre-existing epigenetic switch (Fig. 2). Central to the switch is the latter portion of the pyrimidine biosynthetic pathway, which we postulate includes a cell-fate decision point. Together, these components possess functional and regulatory connectivities that a simple mathematical model shows are sufficient to generate stochastic switching.

## Results

### An Epigenetic Basis for Capsule Switching

The transition between capsulation states in genotype 1B^4^ resembles phase or antigenic variation in pathogens, which is typically underpinned by contingency loci [11]. Initial hypotheses predicted that the mechanism of switching would be mutational, involving either high-frequency gain and loss of the *carB* mutation or amplification and collapse of the *carB* locus. Both hypotheses were rejected (see S1 Text). Attention therefore turned to the likelihood that the *carB* mutation established an epigenetic switch.

In order to identify genes with a role in capsule switching, 1B^4^ was subjected to transposon mutagenesis [12,15]. From a pool of ~69,000 transposon mutants, 157 were deficient in their capacity to switch between capsulation states. While six showed an increase in the proportion of Cap^+^ cells, 151 showed loss (or reduction) of Cap^+^ (S1 Table). Thirty-eight mutants were implicated in biosynthesis of a capsular polymer (see below). Of the remaining 119 mutants, 38 contained insertions in potential regulators of capsule biosynthesis (see below) and 81 contained insertions in genes involved in a wide range of cellular processes, the most prominent of which were transport and central metabolism (22 mutants), cell division and DNA processing (11 mutants), RNA processing (ten mutants), and nucleotide biosynthesis (five mutants).

### Capsule Synthesis Relies on the Expression of a Colanic Acid-Like Polymer

**Fig 3:**
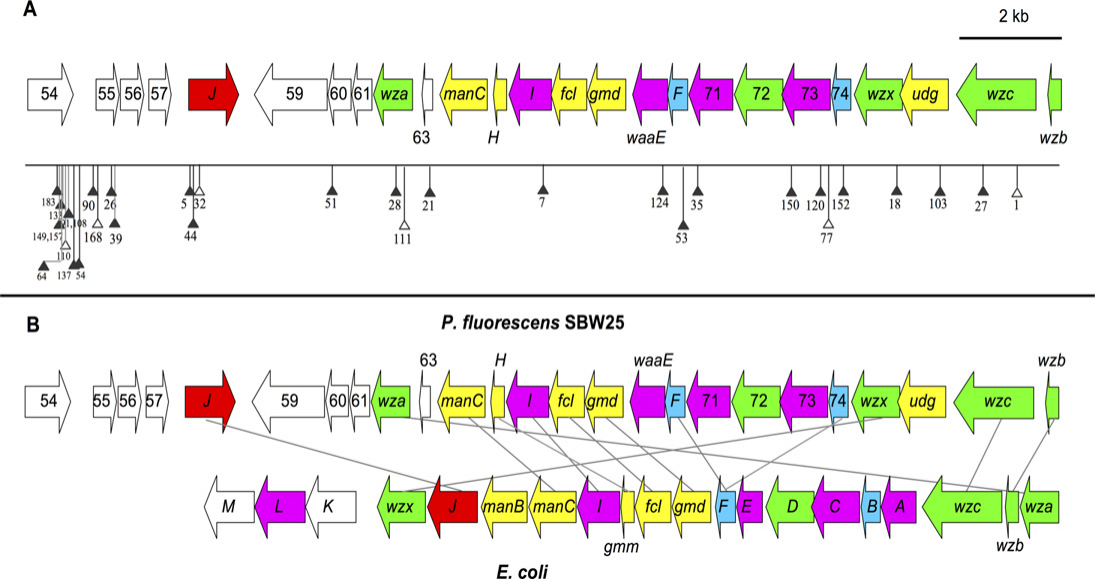
The SBW25 CAP biosynthetic gene cluster. (**A**) Transposon insertions in SBW25 genes *pflu*3658–78 (filled triangles = precise insertion site identified, and unfilled = precise site unknown; numbers relate to S1 Table strain names). See S1 Fig for reactions catalysed by enzymes disrupted by transposon insertion. (**B**) Comparison of the SBW25 and *E*. *coli* K-12 colanic acid biosynthetic loci. The SBW25 locus is predicted to contain five genes involved in CAP precursor biosynthesis (yellow), *wcaJ* (red), four glycosyl transferases (purple), two acetyl transferases (blue), five genes involved in polymerisation and transport (green), and four genes of unknown function (white). Homologous gene products (connected by grey lines) were identified with Basic Local Alignment Search Tool protein (BLASTP, a tool used to search a query protein against a protein database).

Nineteen transposon mutants contained insertions in the *wcaJ-wzb* locus (*pflu3658–3678*), a region of the genome that resembles the colanic acid biosynthetic locus of *Escherichia coli* (Fig. 3) [16]. Deletion of the entire *wcaJ-wzb* locus (giving 1B^4^-Δ*wcaJ-wzb*) produced translucent colonies (Fig. 1E) comprised of Cap^-^ cells. These phenotypes were indistinguishable from those of 19 transposon mutants with insertions in *wcaJ-wzb* (previously reported in [12]).

A further 19 mutants contained insertions in genes encoding enzymes required for biosynthesis of guanosine diphosphate (GDP)–fucose, uridine diphosphate (UDP)–glucose, and UDP—glucuronic acid [12], three of the four building blocks for colanic acid (see S2 Fig) [16]. These include insertions in *galU* (EC 2.7.7.9), *pgi* (EC 5.3.1.9), *pflu5986* (EC 5.4.2.8/5.4.2.2), and *udg* (EC 1.1.1.22) [17]. The usual genes encoding enzymes for biosynthesis of the fourth precursor (UDP-galactose) are absent in SBW25, indicating that the 1B^4^ capsule polymer is structurally different than colanic acid. Indeed, analysis of the monosaccharide composition of extracellular polysaccharide (EPS) in SBW25, 1A^4^, 1B^4^, and two transposon mutants indicates that the 1B^4^ capsule polymer contains six monosaccharides: fucose, glucose, glucuronic acid, galactouronic acid, and two unidentified monosaccharides (S2 Table). Only three of these monosaccharides are present in colanic acid, which consists of repeating units of fucose, galactose, glucose, and glucuronic acid [18].

On the basis of the genetic organisation of the *wcaJ-wzb* locus, the similarity of these enzymes to functionally characterized enzymes from *E*. *coli*, the requirement for colanic acid-like precursors, and the monosaccharide analysis, we conclude that the 1B^4^ capsule polymer is related to, but distinct from, colanic acid. Henceforth, the 1B^4^ capsule polymer will be referred to as the colanic acid-like polymer (CAP).

### Bistable CAP Expression in 1B^4^ Is Controlled at the Transcriptional Level

Epigenetic switches are often underpinned by differences in gene expression. Total mRNA was extracted from SBW25, 1A^4^, 1B^4^-Cap^-^, and 1B^4^-Cap^+^ cells and sequenced. Four pairwise comparisons (SBW25 versus 1A^4^, 1A^4^ versus 1B^4^-Cap^-^, 1A^4^ versus 1B^4^-Cap^+^, and 1B^4^-Cap^-^ versus 1B^4^-Cap^+^) allowed differences in transcription to be detected (S2–S5 Tables). Each comparison revealed differential expression of ~60% of all coding sequences, including expression of genes encoding polymer biosynthetic enzymes, cell surface components, metabolic enzymes, and cell division genes. Of note is that transcription of *wcaJ-wzb*, the CAP biosynthetic locus, was significantly greater in 1B^4^-Cap^+^ than 1A^4^ or 1B^4^-Cap^-^. To corroborate this, a promoterless *lacZ* gene was transcriptionally fused to 1B^4^ *wcaJ*. Growth of 1B^4^-*wcaJ-lacZ* on media containing X-gal gave rise to colonies with blue sectors comprised of predominantly Cap^+^ cells (Fig. 1F), indicating that *wcaJ* transcription is indeed increased in Cap^+^ cells.

The 1B^4^ transposon mutagenesis screen identified three regulators of CAP biosynthetic genes (Pflu4939, BarA-GacA, and Pflu3654-Pflu3657; S1 Table). The first of these is Pflu4939, a probable transcriptional regulator with homology to MvaT, a global regulator of virulence gene expression in other pseudomonads [19]. Three independent transposon insertions in *pflu4939* (and the ~10 bp immediately upstream) led to increased capsule production, suggesting that Pflu4939 is a negative regulator of CAP biosynthetic gene transcription. Conversely, insertions in loci encoding the other two regulatory systems caused elimination or reduction of capsule formation, indicating that these are activators of CAP biosynthetic gene transcription.

The second regulatory pathway, identified by 24 transposon insertions, is the BarA-GacA two-component signal transduction relay system. BarA-GacA is known to regulate a broad range of virulence and stress response systems in various gram-negative bacteria [20]. The third regulatory system is identified by 14 insertions in *pflu3654-pflu3657*, a genomic region directly upstream of the CAP biosynthetic genes (see Fig. 3), most of which has no significant database matches. Notably, when a transcriptional fusion of *gfpmut3* and the genomic region upstream of *pflu3655-pflu3657* was inserted into the 1B^4^ genome, expression of green fluorescent protein (GFP) in Cap^+^ cells was observed (see S2 Fig and S2 Text). This demonstrates that this region contains a promoter sequence transcribed in response to the capsule ON signal.

None of the above regulators bears homology to the Rcs phosphorelay that controls colanic acid gene transcription in the *Escherichia*, *Erwinia*, *Klebsiella*, and *Rhizobium* genera [21]. Indeed, a search of the SBW25 genome using translated Basic Local Alignment Search Tool nucleotides (TBLASTN, a tool used to search a query protein against a translated nucleotide database) revealed no loci with homology to *E*. *coli rcsA*.

### The Pyrimidine Biosynthetic Pathway Links *carB* and the CAP Biosynthetic Genes

Thus far, phenotypic observation of 1B^4^ colonies, combined with the results of transposon mutagenesis and transcription analyses, indicates colony switching to be the result of bistable CAP expression, which is at least partially effected at the transcriptional level. To understand the epigenetic events by which the *carB* mutation influences CAP expression, we next studied the pyrimidine biosynthetic pathway, which provides a molecular link between *carB* and CAP (Fig. 2). In brief, CarB is the product of the second gene of the *carAB* operon, which encodes the two subunits of carbamoyl phosphate synthetase (CPSase, EC 6.3.5.5). The product of CPSase, carbamoyl phosphate (CP), is the precursor for synthesis of arginine and pyrimidines. The pyrimidine biosynthetic pathway concludes with synthesis of UTP, which is further metabolised by PyrG (to produce cytidine triphosphate [CTP] for DNA synthesis) and/or GalU (to produce UDP-glucose, a precursor of CAP biosynthesis).

**Fig 4:**
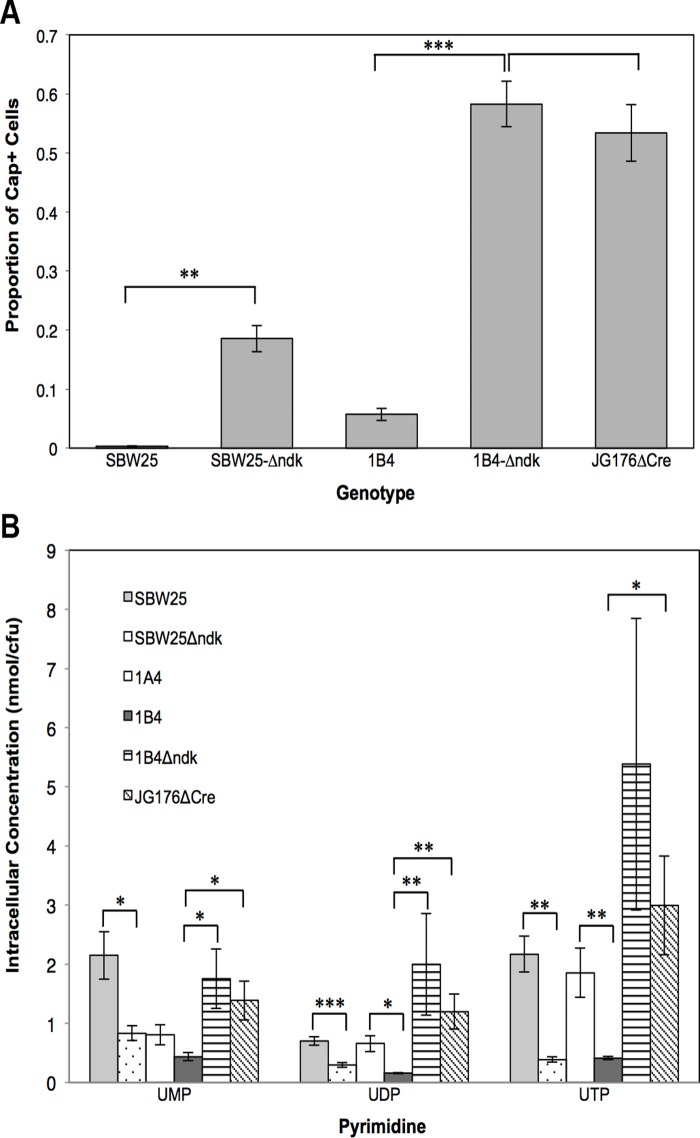
Deleterious mutations in *carB* and *ndk* affect capsulation and intracellular concentrations of pyrimidine intermediates. The *ndk* gene was deleted from SBW25 and 1B^4^ using genetic engineering techniques, giving SBW25-Δ*ndk* and 1B^4^-Δ*ndk*, and the effects on capsule switching and pyrimidine pathway intermediates were determined. Bars are means of five replicates, and error bars show +/- 1 SE. (**A**) Capsulation levels in evolved and engineered genotypes. Deletion of *ndk* caused an increase in capsulation in SBW25 (i.e., in the absence of a *carB* mutation; Wilcoxon rank-sum test *p* < 0.01) and 1B^4^ (i.e., in the presence of c2020t *carB*; Welch two-sample *t* test; *p* < 0.001). No significant difference in capsulation levels was found between 1B^4^-Δ*ndk* and the *ndk* transposon mutant, JG176ΔCre (two-sample *t* test, *p* > 0.5). (**B**) Uridine monophosphate (UMP), UDP, and UTP levels were measured in the indicated genotypes (see S7 Table and S8 Table for independent measurements and measurement of additional metabolites and genotypes). The c2020t *carB* mutation significantly reduced concentrations of UDP and UTP (two-sample *t* tests for 1A^4^ versus 1B^4^, *p* < 0.05). Similarly, deletion of *ndk* significantly reduced concentrations of UMP, UDP, and UTP (two-sample *t* tests for SBW25 versus SBW25-Δ*ndk*, *p* < 0.05). Coexistence of c2020t *carB* and the *ndk* deletion resulted in an increase in UMP, UDP, and UTP (two-sample *t* tests or Wilcoxon rank-sum tests for 1B^4^ versus 1B^4^-Δ*ndk* and/or JG176ΔCre, *p* < 0.05). No significant differences in UMP, UDP, or UTP concentrations were found between 1B^4^-Δ*ndk* and the *ndk* transposon mutant, JG176ΔCre (two-sample *t* tests or Wilcoxon rank-sum tests, *p* > 0.05).

The importance of the pyrimidine biosynthetic pathway in 1B^4^ capsule switching is corroborated by the isolation of a transposon mutant (JG176) carrying an insertion in one of the pathway genes, *ndk* (encoding nucleoside diphosphate kinase, EC 2.7.4.6). This mutant is intriguing because it generates uniformly opaque colonies—a rare phenotype in the mutagenesis screen. Cre-mediated excision of the transposon left a 189 bp inactivational insertion in *ndk*, creating JG176ΔCre and eliminating the possibility of polar effects. The mutant showed elevated levels of capsulation compared to 1B^4^ (Fig. 4A). A similar increase was observed upon deletion of the entire *ndk* reading frame from 1B^4^ (1B^4^-Δ*ndk*; Fig. 4A). Deletion of *ndk* from wild type also resulted in an increase in capsulation (SBW25-Δ*ndk*; Fig. 4A), demonstrating that a reduction in *ndk* activity plays a role in capsule bistability; hypercapsulation as a result of *ndk* inactivation indicates that capsule production is a response to perturbations in the supply of nucleotide precursors. Notably, SBW25-Δ*ndk* has a lower capsulation level than 1B^4^-Δ*ndk* (*p* < 0.01), highlighting that multiple mutations in the pyrimidine pathway can have additive effects on capsule bistability.

Given that production of nucleoside triphosphates (NTPs) is an essential intracellular process, the viability of the *ndk* deletion strains indicates that SBW25 possesses at least one alternative enzyme with nucleoside diphosphate kinase (NDK) activity; adenylate kinase [22], pyruvate kinase [23], and polyphosphate kinase [24] have been shown to function as alternative NDKs in other species. While each enzyme showing NDK activity uses UDP, CDP, GDP, and adenosine diphosphate (ADP) as substrates for NTP production, differing affinities of the enzymes for each substrate can carry large consequences for intracellular UTP, CTP, GTP, and adenosine triphosphate (ATP) concentrations [23]. Changing NDK activity through *ndk* deletion is therefore expected to have unpredictable consequences for all nucleoside diphosphate (NDP) and NTP pools in SBW25 and 1B^4^ cells.

### The *carB* Mutation Reduces Concentrations of Pyrimidine Biosynthetic Intermediates

Reduced growth of 1B^4^ compared to that of the immediate precursor genotype (1A^4^) indicates that the *carB* mutation causes a reduction in CPSase function (S3 Fig). In line with this, direct measures of pyrimidine intermediates in 1A^4^ and 1B^4^ demonstrated statistically significant reductions in intracellular concentrations of UDP and UTP as a consequence of the c2020t *carB* mutation (Fig. 4B, S7 Table). Similar measurements in SBW25 and SBW25-Δ*ndk* revealed that deletion of *ndk* leads to significant reductions in UMP, UDP, and UTP (Fig. 4B, S7 Table). Thus, we conclude that function-reducing mutations in pyrimidine biosynthetic genes lead to reductions in pyrimidine pathway intermediates (Fig. 4B), which in turn lead to increases in capsulation (Fig. 4A). (For concentrations of other intracellular metabolic intermediates in a range of genotypes, see S8 Table.)

Given the above results, it is likely that the greater the loss in pathway functionality, the greater the reduction in pyrimidine pathway intermediates. However, genotypes containing a mutation in both *carB* and *ndk* (1B^4^-Δ*ndk* and JG176ΔCre) showed an increase in UMP, UDP, and UTP compared to 1B^4^ (Fig. 4B), despite relative capsulation increases (Fig. 4A). This result was confirmed by a second, independent set of measurements performed using an alternative protocol (see S8 Table). The observations in these double-mutant genotypes are likely indicative of the complexity of regulatory systems underpinning central metabolism (see also Results section above) [25].

### Addition of Exogenous Uracil Affects the Proportion of Capsulated Cells

**Fig 5:**
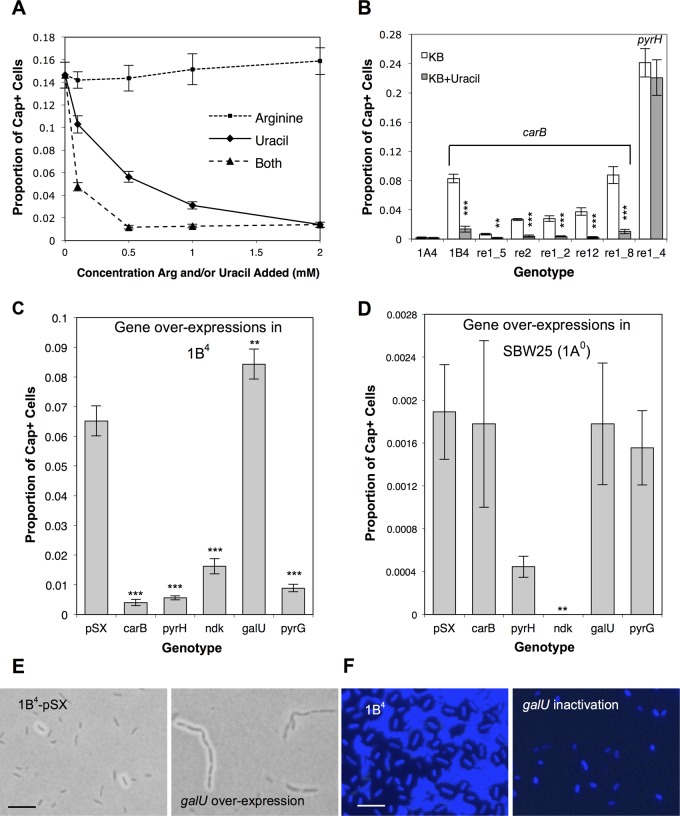
Changes in pyrimidine biosynthetic pathway flux alter proportion of Cap^+^ cells. (**A**) Addition of 2 mM uracil to 1B^4^ growth medium causes a reduction in the proportion of Cap^+^ cells (two-sample *t* test, *p* < 0.001); addition of 2 mM arginine has no effect (two-sample *t* test, *p* > 0.1). The addition of both uracil and arginine reduces capsulation levels below those seen with uracil alone, possibly as a result of complex regulatory interactions. Each point is the mean of five replicates, and error bars show +/- 1 SE. Asterisks indicate a significant difference between each genotype in the presence and absence of uracil. (**B**) Seven independently isolated switcher genotypes (see Table 1) show varying proportions of Cap^+^ cells. Addition of 2 mM uracil significantly reduces Cap^+^ levels in *carB* switchers (two-sample *t* tests, *p* < 0.05) but has no effect on Cap^+^ in the *pyrH* switcher (Re1_4, *p* > 0.1) or 1A^4^ (*p* > 0.1). Bars are means of five replicates, and error bars show +/- 1 SE. (**C–D**) The proportion of Cap^+^ cells when overexpressing *carB*, *pyrH*, *ndk*, *galU*, or *pyrG* in 1B^4^ (C) and SBW25 (D). “pSX” bars are means of 18 replicates (six replicates from each of three biological replicates), while others are means of nine or six replicates (three replicates from each of two or three biological replicates). Significance tests are for differences between the relevant genotype and empty pSX. (**E**) Light microscope image of 1B^4^ + pSX (left) and 1B^4^ + pSX-*galU* (right). Overexpression of *galU* results in cell chains. Bar ~5 μm. The saturation of the images was altered. (**F**) Fluorescence microscope image of 1B^4^ (left) and JG49 (right) cells stained with Fluorescent Brightener 28 (cellulose binding dye). Inactivation of *galU* gives short cells. Bar ~5 μm. The exposure of the second image was altered.

Results to this point have demonstrated that the *carB* mutation causes (i) a reduction in concentrations of pyrimidine intermediates and (ii) capsule switching. Next, we investigated whether reduction of one or more pyrimidine intermediates causes (rather than merely correlates with) capsule switching. If the relationship were causal, then switching should be reduced or eliminated by restoration of the concentration of relevant pyrimidine intermediates to ancestral levels. Thus, the effect of adding increasing amounts of exogenous uracil—which is taken up by cells and enters the pyrimidine biosynthetic pathway as UMP (see Fig. 2)—on capsule switching was investigated. Uracil addition gradually decreased capsule switching, and a concentration of 2 mM uracil significantly reduced the proportion of capsulated cells (two-sample *t* test, *p* < 0.001; Fig. 5A). By way of control, the effect of adding increasing amounts of exogenous arginine, the biosynthesis of which also depends on *carB*, was investigated and found to have no effect on the proportion of capsulated cells (Fig. 5A). Additionally, quantification of the effect of adding guanine hydrochloride on capsulation revealed no direct role for purines—intracellular levels of which are tightly coordinated with pyrimidines [14]—in capsule switching (S3 Text).

Together, the above findings not only demonstrate a causal relationship between reduction of pyrimidine biosynthetic intermediates and capsule switching, but they also show that the switching phenotype can be eliminated through provision of an intermediate (UMP) several enzymatic steps downstream of *carB*. This indicates that capsule switching is dependent on reduced availability of UMP and/or downstream products.

### A Mutation in *pyrH* Causes Capsule Switching

Work to this point has shown that the likelihood of cells becoming capsulated is negatively related to the supply of pyrimidine pathway intermediates, particularly downstream of UMP. In principle, supply-reducing mutations elsewhere in the pyrimidine pathway should effect a similar reduction in pyrimidine concentrations, leading to switching. In order to investigate whether mutations in genes other than *carB* can give rise to capsule switching, an evolution experiment was performed in which we sought to evolve new switching genotypes from 1A^4^. Each of these genotypes was expected to contain the first eight mutations of the evolutionary series (Fig. 1A) plus an independently evolved switch-causing mutation. A set of 36 independent replicate populations of 1A^4^ (the nonswitching predecessor of 1B^4^) was subjected to one round of selection under the regime that delivered 1B^4^. At the conclusion of this “re-evolution” experiment, cells from each population were plated on agar and screened for the presence of rapidly sectoring colonies.

From 36 replicates, six populations produced switchers. A capsule counting assay showed that each switcher genotype produced populations of cells with distinctly different ratios of Cap^+^ to Cap^-^ cells (Fig. 5B), suggesting that the underlying mutations had quantitatively different effects on concentrations of pyrimidine intermediates and the likelihood of switching. Indeed, analysis of intracellular metabolite levels in two of the re-evolved switchers, Re1_4 and Re1_5, revealed that the switch-causing mutations in these genotypes cause a multitude of metabolic effects (S8 Table), including alteration of the concentration of pyrimidine intermediates.

In order to see whether the switch-causing mutations lay upstream or downstream of the entry point of uracil, the effect of uracil supplementation on the frequency of switching was examined. Five of the six re-evolved switchers showed a response to uracil that was typical of 1B^4^; however, the sixth (Re1_4) was unresponsive (Fig. 5B). On sequencing the *carB* gene, each of the five uracil-responsive switchers was found to contain a *carB* mutation (Table 1). Re1_4 did not contain a *carB* mutation. Genome resequencing of Re1_4 revealed the final mutation as a single, nonsynonymous change in *pyrH* (R123C). PyrH encodes uridylate kinase (EC 2.7.4.22), the enzyme responsible for the phosphorylation of UMP to UDP. As the UDP/UMP ratio of Re1_4 was decreased 28-fold relative to that of 1A^4^ (see S8 Table), this mutation was deleterious to PyrH function. Given that *pyrH* is six genes along the pyrimidine pathway from *carB*, this finding reduced the number of genes and regulatory connections under consideration: from this point on, attention focused on *pyrH* and downstream genes.

**Table 1:** Mutations causing capsule switching.

Genotype	*pflu*	Gene Name	Nucleotide Change^1^	Amino Acid Change
1B^4^	5265	*carB*	c2020t	R674C
Re1_5	5265	*carB*	c836t	T291I
Re2	5265	*carB*	a2477g	N826S
Re1_2	5265	*carB*	c431t	P144L
Re12	5265	*carB*	g695a	C232Y
Re1_8	5265	*carB*	c2020t	R674C
Re1_4	1272	*pyrH*	c331t	R123C

^1^The switch-causing mutations in 1B^4^ and Re1_8 are identical.

### Overexpression of Pyrimidine Biosynthetic Genes Affects Capsule Switching

To define a set of genes necessary for switching, the expression of each gene downstream of *pyrH* was manipulated. We reasoned that manipulations of any candidate gene that led to a change in the behaviour of the switch indicated the involvement of that component in the circuit. Thus, *carB*, *pyrH*, *ndk*, *galU*, and *pyrG* were overexpressed in turn in 1B^4^, and the ratio of Cap^+^ to Cap^-^ cells determined for each (Fig. 5C). Although no longer a central focus, *carB* was included in the overexpression set by way of positive control; given that 1B^4^ switching results from a loss of *carB* function, successful overexpression of wild-type *carB* is expected to reduce capsulation.

Identifying the point in the pyrimidine pathway at which the switch decision occurs is central to understanding the switch mechanism. Logic suggests this is likely to be the UTP bifurcation point (see Fig. 2), and the results shown in Fig. 5C are consistent with this prediction. First, overexpression of *carB*, *pyrH*, and *ndk* (the three enzymes immediately prior to the bifurcation) significantly lowered 1B^4^ capsulation levels, indicating that sufficiency prior to the UTP bifurcation directs pathway resources away from capsulation. Second, overexpression of PyrG and GalU (two enzymes immediately beyond the UTP decision point) had opposing effects on 1B^4^ capsulation: *pyrG* overexpression lowered capsulation, while *galU* overexpression increased capsulation. The ability to both decrease and increase capsulation through manipulation of these genes demonstrates that these components encompass key elements of the switch.

In addition to increasing 1B^4^ capsulation levels, *galU* overexpression led to cell chains. Each chain, composed of 2–10 cells, was exclusively Cap^+^ or Cap^-^ (Fig. 5E). This is in contrast to the phenotype of the Cap^-^ *galU* transposon mutant (JG114ΔCre, S1 Table), which is low in UDP-glucose (S8 Table) and produces short cells (Fig. 5F). The fact that an increase in GalU inhibits cell division (producing cell chains) and a reduction in GalU stimulates cell division (producing short cells) indicates that GalU, or downstream pathway components, play a role in regulation of cell division. Indeed, such a finding has recently been reported in *E*. *coli* [26] and *Bacillus subtilis* [27], in which UDP-glucose appears to function as a “metabolic sensor” that couples nutrient availability to cell division. Introduction of the *carB* mutation from SBW25 to *E*. *coli* B REL606 did not generate any obvious cellular- or colony-level phenotypic switching (S4 Fig).

### Capsule Expression Is Costly, and Switching Depends on Population Size

In order to directly observe capsule switching events, a genotype carrying a transcriptional fusion of *gfpmut3* and the region directly upstream of *pflu3655*—a probable regulator of CAP expression (see Fig. 3 and S1 Table)—was constructed in 1B^4^ (see S2 Text). Microscopic analysis of the resulting genotype, 1B^4^-CAP-GFP, revealed a 100% correlation between GFP and capsule expression; Cap^+^ 1B^4^-CAP-GFP cells fluoresce, while Cap^-^ cells do not (S2 Fig).

The growth of single 1B^4^-CAP-GFP cells into microcolonies was observed microscopically. First, the development of microcolonies founded by noncapsulated cells was examined (S2A Fig). The switch from Cap^-^ to Cap^+^ was found to be dependent on population size; Cap^-^ to Cap^+^ switch events were only observed once a population size of 1870 ± 660 cells was exceeded (*n* = 58 microcolonies). In accordance with this, the distribution of the population size at which the first switch event is observed is Gaussian (S2B Fig). This is in contrast to a Poisson distribution, which would have supported a random process independent of population size. However, once a minimum threshold population size is reached, Cap^-^ cells switch to Cap^+^ at random, and, thereafter, the expression of CAP genes is heritable (S1 Video). In contrast to Cap^-^ founder cells, Cap^+^ founder cells were observed to systematically switch to the Cap^-^ form within a very short time frame (S2 Video).

To investigate the cost of capsule production, we measured the growth rate of Cap^-^ and Cap^+^ cells using image analysis. Using a conservative estimate, Cap^+^ cells were found to grow more slowly (growth rate of 0.0113 min^-1^ and SE = 2.5 x 10^-4^ min^-1^, or one division every ~61.3 min; *n* = 39) than Cap^-^ cells (growth rate of 0.0116 min^-1^ and SE = 6.10 x 10^-5^ min^-1^, or one division every ~59.8 min; *n* = 17). This demonstrates a cost for capsule synthesis of ~3%.

### An Epigenetic Model for Capsule Switching: The Growth-Capsulation Model

While genetic and biochemical data define a minimal set of components involved in capsule switching, specific molecular details remain unknown. Nonetheless, we present a simple mathematical model in order to show that the bifurcation of the UTP pool into CTP and UDP-glucose synthesis can, with a minimal set of plausible assumptions regarding regulation (outlined in S5 Fig), give rise to capsule switching. For simplicity, the model assumes a molecule—hereafter referred to as UXP—whose level is proportional to UTP as the central signal molecule for capsule biosynthesis. In reality, the signal could be any one, or a combination, of many possibilities (e.g., pyrimidine pathway intermediates, biosynthetic enzymes, and transcription factors).

**Fig 6:**
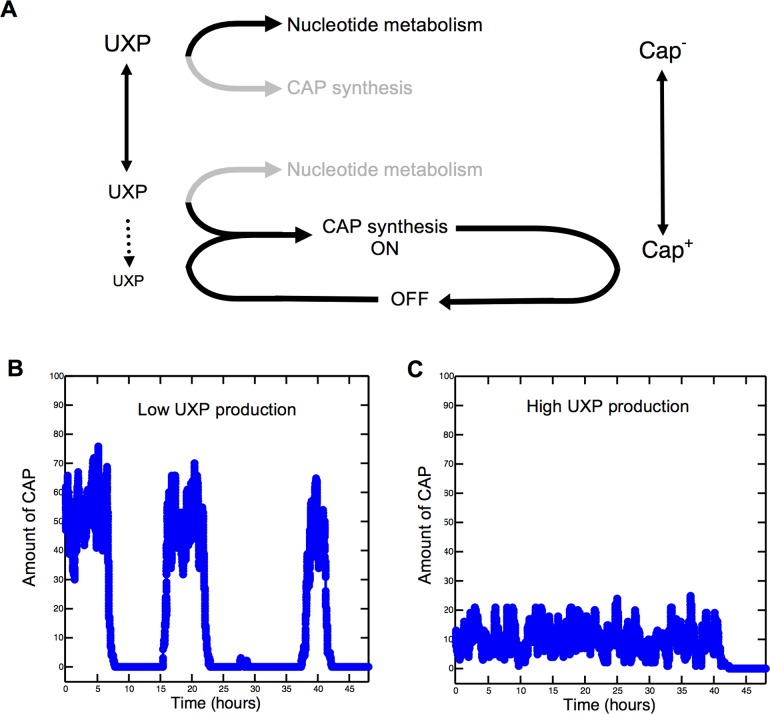
The growth-capsulation model for stochastic capsule switching. (**A**) A schematic model for capsule switching. Central to the switch is the bifurcation of the UTP pool into nucleotide metabolism (via PyrG) and CAP synthesis (via GalU). When UXP (a signal molecule proportional to UTP) levels are high, cells commit resources to nucleotide metabolism, and expression of the GalU branch is basal. As UXP levels fall, the likelihood that CAP synthesis is activated increases because of the accumulation of a positive regulator sensitive to UXP levels. Once a cell commits to CAP synthesis, nucleotide metabolism slows, and continued CAP synthesis positively feeds back to maintain low UXP levels. To enable a return to Cap^-^, we model negative feedback via a repressor activated concomitantly with CAP synthesis. The length of time cells remain Cap^+^ depends on many factors that are each subject to molecular noise. This introduces stochasticity to the system; we model this using the Gillespie algorithm (see Results text for more detail). (**B**) A simulation of the model showing that low UXP production can induce switching through changes in CAP expression. Low UXP promotes CAP expression through increased availability of a presumed transcriptional activator of CAP biosynthetic genes. Cap^+^ persists until the CAP repressor, induced by CAP, is synthesized and outcompetes the activator. Bound repressor causes CAP mRNA levels to fall because of halted synthesis and degradation. Cap^-^ persists until repressor concentration decreases and the DNA binding site is free. UXP is low, and the cycle repeats. See also S5 Fig, S1 Code, and S2 Code. (**C**) Same as B but with elevated UXP production. The positive regulator-repressor system is not utilized; thus, CAP expression remains basal.

Shown in Fig. 6A, the growth-capsulation model postulates that capsule bistability arises as a result of differential utilization of the UTP pool at the bifurcation point (see Fig. 2): Cap^-^ prevails when UTP is preferentially channelled by PyrG into DNA metabolism (and ultimately cell division), while Cap^+^ results from utilization of UTP by GalU for capsule biosynthesis. The likelihood of a cell entering the Cap^+^ state increases as intracellular UXP levels decline. This we model as a positive regulator of CAP biosynthesis whose activity is inversely proportional to the availability of UXP. For example, the regulator may have high affinity for UXP, which ensures that at high UXP concentrations the regulator is inactive. However, as UXP concentrations decline, the regulator becomes free, resulting in high-level expression of CAP biosynthetic genes. As previously noted, the 1B^4^ transposon mutagenesis screen identified two genetic loci encoding transcriptional regulators required for maximal CAP biosynthetic gene transcription: *pflu3655-pflu3657* and *barA/gacA* (S1 Table). It is plausible that the activity of one of these regulators is sensitive to changes in concentrations of pyrimidine intermediates.

Experimental observations show that the Cap^+^ state is heritable (S1 Video, [12]). This “phenotypic memory” depends on maintenance of low UXP levels, which we model as a positive feedback loop whereby the synthesis of CAP draws UTP into the capsule production. Although UDP from UDP-glucose is recycled back through UMP, commitment to CAP synthesis ensures a reduction of the UXP pool. Duration of the Cap^+^ state depends on molecular noise inherent in the mechanism of regulation (e.g., uneven splitting of intracellular molecules upon cell division, strength of positive regulator-promoter binding, and rate of CAP degradation). To model this, interactions among components were simulated using a stochastic simulation algorithm [28]. For a switch to Cap^-^, UTP utilization must shift from CAP biosynthesis in favour of nucleotide metabolism. The likelihood of this occurring increases with increasing UXP levels. We model this as a time-delayed, autoregulatory feedback loop that restricts the activity period of capsule synthesis. For example, CAP may inhibit its own biosynthesis.

In order to demonstrate that the components of the model are capable of generating switching, we used the chemical equations in S5 Fig to simulate changing concentrations of CAP in a single cell over a 48-h period. Further, to investigate the effect of UXP levels on capsulation state, the simulation was performed with differing UXP production rates. Fig. 6B shows that at low UXP production rates, a cell typically has two widely separated levels of CAP expression over 48 h, representing the Cap^+^ (higher) and Cap^-^ (lower) states. Notably, both the high and low CAP levels are maintained—in a probabilistic manner—over several cell divisions (i.e., are heritable). This phenotypic dichotomy is typically eliminated at higher UXP production rates; Fig. 6C shows CAP expression maintained at a relatively constant basal level. Further examples of simulated CAP expression at high, low, and intermediate UXP levels are provided in S6 Fig.

This simple model demonstrates stochastic capsule switching in which both states persist for a duration that is long enough to allow epigenetic heritability (sufficient to generate colonies that are visibly Cap^+^ or Cap^-^) but short enough to enable each state to give rise to the other. Furthermore, UXP concentration in the system determines the probability of switching. While the model relies on stochasticity in biochemical reactions, it is expected that other biological factors—such as cell division, which appears to be linked to GalU (and thus CAP) levels—will also contribute to heterogeneous populations [29].

In addition to simulating stochastic switching, the model also encompasses a circuitry (of reactions, reaction rates, and number of molecules) that allows the level of stochasticity to be tuned. To demonstrate this, the 23 reaction rates (determined by the equations in S5 Fig) were altered and the resulting time spent in Cap^-^ and Cap^+^ states determined. The value of each reaction rate, one at a time, was decreased by a factor of ten, and the simulation run ten times. This process was then repeated but with a 10-fold increase in each rate. The fraction of time for which the network exhibited the Cap^-^ phenotype was determined under all 47 sets of conditions. The results (shown in S9 Table and S7 Fig.) reveal phenotypes that are Cap^-^ between 11.6% and 90.4% of the time, indicating tuning over a wide range of reaction rates. Thus, while certain specific molecular details of the switch remain to be determined, the growth-capsulation model is consistent with experimental observations to date.

### A Test of the Model: Capsule Switching in SBW25 (1A^0^)

The above model postulates that all machinery necessary for switching is present in the ancestral SBW25 genome but that distance from the Cap^+^ “threshold”—due to sufficiency of UXP under normal laboratory culture—means that colonies appear uniformly translucent. Upon discovery of a mutation that reduces flux through the pyrimidine pathway, the model predicts that the population comes closer to the Cap^+^ “threshold” and thus the possibility that extrinsic noise places more of the population above the threshold. Accordingly, there is reason to expect the occasional occurrence of Cap^+^ cells in colonies of the ancestral type that are otherwise visibly translucent. Indeed, a capsule counting assay revealed the proportion of capsulated cells in SBW25 populations to be 0.0016 (~1 capsule per 625 cells) compared with 0.084 (~1 in 12 cells) in 1B^4^ populations (Fig. 1D).

If the capsulated cells observed in SBW25 and 1B^4^ are a result of the same switch mechanism, then it is expected that Cap^+^ cells of both genotypes will respond to manipulations of the pyrimidine biosynthetic pathway in a similar manner. Thus, as was done for 1B^4^ (Fig. 5C), *carB*, *pyrH*, *ndk*, *galU*, and *pyrG* were each sequentially overexpressed in SBW25. A capsule counting assay performed on each genotype (Fig. 5D) revealed that, as seen for 1B^4^, overexpression of *ndk* resulted in a significant reduction in SBW25 capsulation (no capsules observed in 4,500 cells). No statistically significant change in capsulation was observed for any other gene, possibly a result of the low number of capsulated cells assayed. Taken together, these results demonstrate that, as predicted by the growth-capsulation model, the capsule switch machinery is present and functional in SBW25.

## Discussion

Rapid switching of colony morphology is a striking phenotypic innovation. In bacterial pathogens, such behaviour is reflective of phase variation in which mutable (contingency) loci control the expression of epitopes on the surface of bacterial cells that interact with the host immune response [11,30]. Variable expression of such epitopes among members of a population allows the risk of elimination by the immune system to be spread among offspring, each of which has some chance of avoiding detection. Similarly, evolution of the capacity in 1B^4^ to switch between opaque and translucent colonies enabled this genotype to succeed in the face of fluctuating selection wrought via an experimental regime that mimicked fundamental features of the host immune response [12].

The ancestral *P*. *fluorescens* SBW25 genotype lacks the capacity to switch colony phenotype under standard laboratory conditions. The fact that switching emerged with relative ease (in two independent lines and with few mutations) suggests a predisposition for evolution of this trait. Our initial prediction was to find modification to an existing tract of nucleotides controlling expression of capsule production that increased the likelihood that this tract underwent mutation. We were therefore surprised to find the cause of switching to be a mutation in *carB*. We were further surprised to find that this mutation, when transferred back into the original ancestor (SBW25), was sufficient to generate switching behaviour [12].

While the proximate cause of switching in 1B^4^ is a mutation in *carB*, the ultimate cause resides in interactions among cellular components (genes, enzymatic products, and metabolic intermediates) surrounding the bifurcation of the UTP pool into CTP and UDP-glucose. At any point in a metabolic pathway where a single, inevitably limiting resource is partitioned into two or more components, there must necessarily be some decision-making mechanism in order to allow cells to match changes in substrate availability with cellular demands [31]. The pre-existence of such a decision-making mechanism—one that allows cells to partition the UTP pool preferentially towards DNA metabolism or polysaccharides—is central to the emergence of switching. Our data show that such a switch exists, that it is probabilistic, responsive to the level of pyrimidine pathway intermediates, and determines whether or not cells commit to a normal cell cycle or to a phase during which protective CAP is produced. The net effect of the *carB* mutation is to induce a shortage in the supply of pyrimidine intermediates, thus bringing the population closer to the threshold level at which the switch is likely to be “thrown.”

A decision to enter the Cap^+^ state and thereby use already scarce pyrimidine resources to synthesize a nonessential polymer may at first appear paradoxical. However, pyrimidine nucleotides are not consumed by the capsule biosynthetic process but instead are recycled back into the pyrimidine biosynthetic pathway as UMP (see Fig. 2). Further, the channelling of UTP into CAP precursor biosynthesis ensures that levels of core pyrimidine nucleotides (UMP, UDP, and UTP) are kept low, making cells less likely to enter the Cap^-^ state (in which pyrimidine resources are diminished by splitting between daughter cells). Thus, CAP biosynthesis might be viewed as a mechanism for conserving scarce pyrimidine resources until such a time that intracellular conditions are optimal for cell division.

Although the 1B^4^ *carB* mutation causes visible switching between colony types, it is clear that the underlying switch (and capacity to toggle between capsulation states) exists in the ancestral genotype. Such a cell cycle checkpoint—underpinned by a stochastic switch—is understandable from the perspective of bacterial survival. In natural environments, resources are often limiting. If the supply of essential components (e.g., nucleotides) to complete the cell cycle is insufficient, then it makes little sense to commit to cell division. An alternate strategy would be to reduce growth rate and invest in protective polymers, thus allowing cells to persist with the chance that intracellular nucleotide concentrations might rise, eventually allowing a normal round of cell division. The switch being probabilistic provides organisms with the possibility to hedge their evolutionary bets. This is in line with the fact that a decision to commit to the cell cycle versus polymer production is necessarily based on knowledge of the present state of the environment. However, the present state will not always accurately predict the future state, and the cost of making a wrong decision (i.e., cell division when nucleotides turn out to be insufficient or polymer production when nucleotides turn out to be abundant) is likely to be high. A stochastic switch would ensure that some cells commit to a strategy that is at odds with the current environment on the off chance that in some future state of the environment that strategy is optimal.

In many regards, capsule switching is reminiscent of both the sporulation process in *B*. *subtilis* [32]—a cell-fate decision that has recently been shown to also be dependent on UDP-glucose levels [33]—and the phenotypic switch to bacterial persister cells [8,32]. Capsule bistability also shares features with the *E*. *coli* lactose switch in which expression of *lacZYA* is either ON or OFF, depending on the presence of a metabolized inducer. Theoretical studies show that at high or low inducer levels the population is uniformly *lacZYA* ON or OFF respectively, while at intermediate inducer levels the population bifurcates, giving rise to both steady states [34]. In terms of 1B^4^ colony switching, high pyrimidine levels result in the CAP OFF state (producing uniform Cap^-^ colonies as in SBW25 and 1A^4^), and lower pyrimidine levels result in bistable CAP expression and thus colony bistability. Even lower pyrimidine levels push the population towards a majority CAP ON and opaque colonies (as in the *ndk* transposon mutant, JG176ΔCre).

If our conclusions concerning the existence of a cell cycle checkpoint are correct, then such a checkpoint is likely conserved across many bacteria. Recent work in *E*. *coli* and *B*. *subtilis* has shown that UDP-glucose and downstream polysaccharide biosynthetic enzymes (OpgH and UgtP, respectively) are part of a signal cascade that influences cell-fate decisions in response to nutrient availability [26,27]. At the molecular level, these enzymes bind to FtsZ, inhibiting assembly of the cell division spindle. Further, it has been noted that disruptions in *E*. *coli* UDP-glucose—through inactivation of *pgm* (S1 Fig.)—alter the timing of cell division, resulting in short cells [35,36]. Disruptions in *pgi*, *pgm*, or *galU* showed a similar, short-cell phenotype in 1B^4^ (S1 Table). Further, overexpression of *galU* resulted in long cells in both 1B^4^ and SBW25 (Fig. 5E). The conservation of these effects on cell division strongly indicates that GalU, UDP-glucose, and/or downstream enzymes influence cell division in SBW25. Thus, we propose that UDP-glucose may act not only as a nutrient sensor but also as a nucleotide sensor during progression through the cell cycle.

Evolution works with the building blocks at its disposal, typically via small modifications [37–39]. While the jump in phenotypic space associated with the emergence of colony switching in 1B^4^ is large, the innovation is underpinned by a minor genetic change that has resulted in apparent modification of the threshold for flipping an existing epigenetic switch; selection appears to have taken advantage of molecular noise to generate an adaptive phenotype. Whether such a starting position is typical for the emergence of a classical contingency locus is unclear, but it is possible that phenotypic switches with an epigenetic basis [40,41] are the evolutionary forerunners of genetic switches and possibly even developmentally controlled regulation [42]. Although the molecular bases of 1B^4^ colony switching currently reside in a regulatory circuit deep in central metabolism, subsequent fluctuating selection could conceivably result in the switch being accommodated in a more specific part of the capsule machinery. Indeed, selection against negative pleiotropic effects associated with the defect in *carB* is likely to be a potent force driving such a process.

## Materials and Methods

### Bacterial Strains and Media

Bacterial strain details are provided in S2 Text. Unless otherwise stated, strains were grown for 16–24 h at 28°C in shaken 30 mL glass microcosms containing 6 mL King’s Medium B (KB) [43], with appropriate supplements. Where stated, uracil, L-arginine hydrochloride, and/or guanine hydrochloride (Sigma-Aldrich) were added to the medium. Cells were plated on lysogeny broth (LB) containing 1.5% agar. Antibiotics were used at the following concentrations: tetracycline (10 μg mL^-1^, Tc), kanamycin (100 μg mL^-1^, Km), gentamicin (liquid medium 10 μg mL^-1^, agar plates 20 μg mL^-1^; Gm), and nitrofurantoin (100 μg mL^-1^, NF).

### Microscopy

Cell microscopy was performed using a Zeiss Axiostar Plus light microscope coupled with fluorescence lighting (HBO 50/AC) where required. Fluorescence microscopy samples were incubated for ~16 h on KB plates containing 10 μg mL^-1^ calcofluor (Fluorescent Brightener 28, Sigma-Aldrich). A dissection microscope was used for colony images. Microscopy images were cropped and processed in Preview as indicated in figure legends.

### Time-Lapse Microscopy and Image Analysis

1B^4^ cultures were grown from glycerol stocks (24 h). Cultures were mixed thoroughly, diluted 100 times in fresh KB, and grown overnight again (24 h). Bacteria were then diluted 10^4^ times and plated on a gel pad (1% agarose in KB). The preparation was sealed with a glass coverslip using double-sided tape (Gene Frame, Fischer Scientific). A duct was cut through the pad centre to allow oxygen diffusion. Time-lapse videos of microcolonies were captured in phase contrast with an automated inverted microscope (IX81, Olympus) using a 100x/NA 1.35 objective (Apo-ph1, Olympus). Images were acquired with an Orca-R^2^ CCD camera (Hamamatsu). Fluorescence excitation was achieved with a mercury vapour light source (EXFO X-Cite 120Q). GFP was imaged with a 485(20)/520(28)-nm filter set using a dichroic beam splitter at 500 nm (Semrock). The growth rates of Cap^-^ and Cap^+^ cells were determined by image analysis using MATLAB routines.

### Capsule Counting Assay

For each strain, 3–5 replicate KB microcosms were inoculated from glycerol stocks. After a 24-h incubation and a 1:1000 transfer, the proportion of Cap^+^ cells was determined in each. Cells were diluted, transferred to a microscope slide, and stained with 1:10 diluted India ink, and a cover slip was added. After 1 min, each preparation was photographed under phase contrast 40x or 63x magnification. Capsule expression was recorded manually for 500 cells per preparation (≤100 cells assayed per photograph). Average proportions of Cap^+^ cells were determined and statistical analyses performed. See also S2 Text.

### Gene Deletions and Mutation Construction

Gene deletions were constructed in the SBW25 background by pUIC3-mediated two-step allelic exchange as described elsewhere [44]. The *carB* mutation was constructed in *E*. *coli* REL606 using a pKOV-mediated two-step allelic exchange as described in [45]. For further details of genetic constructs, see S2 Text.

### Construction and Analysis of 1B^4^-*wcaJ-lacZ*

Wild-type *wcaJ* was PCR-amplified and ligated into pUIC3 [46] immediately upstream of promoterless *lacZY* (see S2 Text). The construct was used to transform *E*. *coli* DH5α-λ*pir* and transferred to 1B^4^ via triparental conjugation (with a helper strain carrying pRK2013). A successful transconjugant was purified, giving 1B^4^-*wcaJ-lacZ*. Single 1B^4^-*wcaJ-lacZ* colonies were grown at 28°C for 56 h on an LB+NF+Tc+X-gal (60 μg mL^-1^) plate prior to microscopic analysis.

### Construction and Analysis of Over-expression Genotypes

The *carB*, *pyrH*, *ndk*, *galU*, and *pyrG* genes were PCR-amplified using primers with restriction sites (see S2 Text). Error-free PCR products were ligated into pSX [47]. Alongside empty pSX, each construct was used to transform chemically competent 1B^4^ and SBW25 cells [48]. Successful transformants were selected and purified on LB+Gm plates, and insert presence was checked by PCR. Three independent genotypes were constructed for each gene-strain combination. Capsule counting assays were performed as previously described.

### Transposon Mutagenesis

1B^4^ was subjected to random mutagenesis as described [15]. Approximately 69,000 transposon mutants from 41 independent conjugations were screened on LB+Km, on which 1B^4^ mutants typically form translucent colonies with opaque sectors. In selected strains, the bulk of the transposon was deleted [15], leaving 189 bp at the insertion site (“-ΔCre” genotypes) and eliminating polar effects.

### Isolation and Analysis of Extracellular Polysaccharide (EPS)

Extracellular polysaccharide (EPS) was isolated from SBW25, 1A^4^, 1B^4^, JG176Δcre, and JG114ΔCre. Each genotype was grown on KB agar (28°C, 48–96 h). Cellular material was resuspended in 12 mL of 1 M NaCl to give an OD_600_ of ~3.5, vortexed for 40 min, and centrifuged (30 min, 4,168 *g*). EPS was precipitated from supernatants by the addition of 3 volumes isopropanol. Following resuspension of pelleted (40 min, 4,168 *g*) EPS in 0.5 mM CaCl_2_, RNAse (0.1 mg mL^-1^) and DNAse (1.2 units mL^-1^) were added, and samples incubated at 37°C overnight. The next day, samples were supplemented with citrate buffer (pH 4.8, 50 mM) and, to eliminate any cellulose, treated with cellulase (ICN Biomedicals; 0.15 mg mL^-1^, 2 h at 50°C). After addition of 0.5 mg mL^-1^ Proteinase K, EPS was precipitated by the addition of 3 volumes isopropanol and pelleted (40 min, 4,168 *g*). EPS pellets were dissolved in dH_2_O (50°C, overnight). Finally, samples were dialysed against dH_2_O for 48 h (SnakeSkin dialysis tubing, Thermo Scientific). The Callaghan Research Institute (New Zealand) performed EPS analysis. Samples were freeze-dried and weighed, and a colorimetric total sugar assay was performed in duplicate for each EPS isolation.

### Genome Sequencing of Re1_4

A Re1_4 colony was grown overnight in KB. Cap^+^ and Cap^-^ fractions were separated by centrifugation, and genomic DNA was isolated from each fraction using the CTAB method. Equal quantities of each isolated DNA were mixed, and whole genome resequencing was performed (Illumina; Massey University, New Zealand). Point mutations were identified by aligning 36-bp sequence reads to the SBW25 genome [49] via SOAP (short oligonucleotide alignment program [50]) and ELAND (Illumina). Insertions and deletions were identified by analysing genomic regions with unusual coverage and BLAST (Basic Local Alignment Search Tool) analysis of discarded sequences.

### mRNA-seq Analysis

SBW25, 1A^4^, and 1B^4^ colonies were grown overnight in KB, diluted 1:1000 into 20 mL KB, and incubated for 24 h. Total RNA was harvested from each; for SBW25 and 1A^4^, 100 μL culture was mixed with 900 μL KB, pelleted, and resuspended in 1 mL of RNAlater (Ambion). For 1B^4^, 100 μL was separated into Cap^+^ and Cap^-^ by centrifugation, and each was resuspended in 1 mL RNAlater. All mRNA extractions proceeded using a RiboPure Bacteria Kit (Ambion). Two mRNA preparations from separate colonies were pooled for each strain. Normalized mRNA-seq library preparation, followed by 100-bp single-end sequencing, was performed by the Australian Genome Research Facility (Brisbane, Australia; accession number GSE48900). The DEseq R package [51] was applied to identify differentially expressed genes.

### Intracellular Levels of Pyrimidine Pathway Intermediates

Intracellular nucleotides were extracted and measured by two independent methods. The first used liquid chromatography mass spectrometry (LC-MS) to measure intracellular concentrations of UMP, UDP, and UTP in five replicates of six genotypes (Fig. 4B, S7 Table). For each sample, single colonies were grown in 16-h glycerol-SA [52] cultures (shaking, 26°C), vortexed, diluted 1:100 or 1:1000 in glycerol-SA, and grown for a further 24 h. Cultures were vortexed, diluted in glycerol-SA to give an OD_600_ of 0.1 (total volume 10 mL in 100-mL flask). Flask cultures were grown until the midexponential phase, when a 1-mL sample was rapidly collected by centrifugation (15 sec, 16,000 *g*). Pellets were immediately frozen in liquid nitrogen until further processing. The remaining culture was used to determine the number of cells per sample (cfu mL^-1^). Samples were extracted by cold extraction [53] and submitted to LC-MS, and the data were analysed as previously described [54]. The second method involved radiolabelling followed by thin-layer chromatography and was used to measure 22 metabolites in triplicate across eight genotypes (S8 Table). Cultures were grown exponentially in glycerol-SA with aeration and labelled with [^33^P]-orthophosphate (Perkin-Elmer) from optical density of 0.1 at 600 nm [52]. For determination of nucleotide pools in Cap^+^ and Cap^-^ fractions, cultures were separated by centrifugation and propagated in fresh media for at least three generations before sampling. Charcoal-binding nucleotides were extracted, separated by two-dimensional thin-layer chromatography, and quantified [55,56].

### Re-evolution of Switchers from 1A^4^

Six independent switcher genotypes were isolated from 1A^4^ according to the evolutionary protocol [12]. Each switcher was purified and its *carB* gene sequenced.

### Simulations

Simulations were performed by incorporating the stochastic Gillespie algorithm into a MATLAB program [28] (S5 Fig, S1 Code, and S2 Code). Initial parameter values were assigned randomly (with the proviso that UTP outnumbered activator molecules), and reaction rates were chosen to promote the equivalent of several cell divisions between switch events, reflecting observed heritability. UTP production rates were adjusted from low to high (1,000-fold difference) to simulate the intracellular state of a switcher and ancestral cell, respectively (S6 Fig). Reaction rates were sequentially increased and decreased by a factor of 10 to investigate the role of stochasticity in switching (S7 Fig and S9 Table). Simulations were run for 200,000 sec (55.55 h) before removal of the initial 10,000 sec, allowing escape from any restrictive initial conditions.

### Statistical Analyses

To detect differences in Cap^+^ levels or nucleotide concentrations between two strains, two-sample *t* tests (parametric or Welch) or, where normality assumptions were violated, Wilcoxon rank-sum tests were applied. To detect differences in Cap^+^ levels across three strains (while testing overexpression genotypes), one-way ANOVA or, where normality assumptions were violated, Kruskal Wallis tests were used. All analyses were performed in R [57]. On the graphs, * = 0.05 < *p* < 0.01, ** = 0.01 < *p* < 0.001, and *** = *p* < 0.001.

## Supporting Information

S1 Code MATLAB code simulating CAP expression in single cells over time. The complete code for the stochastic growth-capsulation model presented in the main Results, S5 Fig, and S6 Fig. The code contains details of initial conditions, reaction rates, parameters, and the simulations presented in Fig. 6B and 6C. UXP level can be set to low (0.003, switchers), intermediate (0.3) or high (3, ancestors). See also S2 Code.
(M) Click here for additional data file.

S2 Code Excel code simulating CAP expression in single cells over time. Identical to S1 Code but in excel format.
(XLS)

S1 Data Raw data for Fig. 1D. (XLS)

S2 Data Raw data for Fig. 4A. (XLS)

S3 Data Raw data for Fig. 4B. (XLSX)

S4 Data Raw data for Fig. 5A. (XLS)

S5 Data Raw data for Fig. 5B. (XLS)

S6 Data Raw data for Fig. 5C and Fig. 5D. (XLS)

S7 Data Raw data for S3 Fig. (XLSX)

S1 Fig SBW25 pathways for biosynthesis of capsule polymer building blocks. Pathways for the biosynthesis of CAP building blocks in SBW25. Genes encoding enzymes are shown by *pflu* number (and name where appropriate; red = recorded transposon mutant [see S1 Table and Fig. 3]). Corresponding *E*. *coli* K-12 gene names are shown in grey in parentheses [58]. The *E*. *coli* UDP-galactose biosynthetic pathway (shown in grey on right) is absent in SBW25.
(TIFF)

S2 Fig Live observation of capsule switching in 1B^4^-CAP-GFP. (**A**) Images from a time-lapse video of a single Cap^-^ 1B^4^-CAP-GFP cell growing into a microcolony. At each of four time points over ~15 h, a phase-contrast image (top) and fluorescence image (bottom, Cap^+^ cells glow green) were captured. (**B**) Fit of the Gaussian distribution for the population size at which capsule switching occurs. (**C**) A comparison of 1B^4^-CAP-GFP cells under fluorescence imaging (left) and stained with India ink (right) shows that Cap^+^ cells reliably fluoresce, while Cap^-^ cells (indicated by arrows) reliably do not.
(TIFF)

S3 Fig SBW25 and 1B^4^ growth in minimal (M9) and supplemented media. Overnight cell cultures of SBW25 (**A**) and 1B^4^ (**B**) were produced from glycerol stocks in shaking KB microcosms. A 1-mL aliquot of each was washed and resuspended in M9 medium to a common cell density (based on OD_600_ of the KB culture). 2-μL aliquots of resuspension were used to inoculate 148 μL of appropriate fresh medium in wells of a 96-well plate. Four types of media were used: M9 (blue lines on graphs), M9 + arginine (0.6 mM, pink lines), M9 + uracil (1 mM, orange lines), and M9 + arginine + uracil (green lines). Each of the eight genotype-medium combinations was replicated five times, and the 16 wells were used as media controls. The OD_600_ of each well was measured at 5-min intervals for 50 h, with 5 sec shaking prior to each read (using a VERSA_max_ plate reader). Hourly time-point data was plotted (data points are means of 5 replicates +/- 1 SE). While 1B^4^ grows more slowly than SBW25 in M9, 1B^4^ is not an auxotroph. The 1B^4^ growth rate deficiency is partially alleviated by addition of arginine, an effect that is increased by the addition of both arginine and uracil. The addition of uracil alone results in an even slower 1B^4^ growth rate than observed in M9. It is likely that this result reflects the complex regulatory systems governing CPSase biosynthesis and activity; pyrimidines have been shown to repress the transcription and activity of *E*. *coli* CPSase [59,60].
(TIFF)

S4 Fig Construction of the *carB* mutation in *E*. *coli* does not cause capsule switching. A *carB* mutation was constructed in *E*. *coli* B REL606. The mutation constructed was c2023t, the equivalent to the SBW25 c2020t *carB* mutation (**A**; a section of SBW25 *carB* aligned with that of REL606, base of interest highlighted in red). Phenotypic analysis of the constructed genotype (REL606-carB*), together with REL606-*carB*wt (containing wild-type *carB*, isolated from the same procedure as REL606-carB*) and 1B^4^, revealed no sign of switching at either the cellular or colony level in the constructed genotype (**B**). Phenotypic assays were conducted at three temperatures (26°C, 30°C, and 37°C) because CAP expression in 1B^4^ was found to be extremely sensitive to temperature, showing no expression over 28°C. Cells of each genotype were grown in LB medium for ~16 h and then transferred to fresh LB medium for ~24 h. Colonies were grown from LB cultures on LB agar for 24 (37°C) or 48 (30°C and 26°C) h.
(TIFF)

S5 Fig Chemical equations defining intracellular processes and conditions surrounding the growth-capsulation model. The equations describe intracellular conditions influencing the capsule switch decision (Fig. 6), demonstrating that a minimal set of components can theoretically give rise to switching. All components and interactions could be substituted so long as low UXP (signal molecule) leads to a bias in UTP utilization towards CAP biosynthesis. The system positive regulator (activator) remains constant for simplicity. (**A**) Describes processes affecting concentrations of the signal molecule, UXP. In the actual biological system, UXP production requires a multistep pathway; for simplicity, we model it as a constant rate (Equation A1). There are two branches for UTP utilization (Fig. 2); in the model, CAP synthesis is regulated solely by CAP (equation A2), and nucleotide synthesis is regulated solely by PyrG (Equation A3). To bias UTP utilization towards CAP under low UXP, the model assumes that UXP sequesters a transcriptional activator for the CAP biosynthetic genes (Equation A4). CA = CAP. (**B**) Encapsulates intracellular processes controlling the transcription of CAP biosynthetic genes. The primary purpose is to enable low UXP to signal CAP production. poly = DNA polymerase, DNA_ca_ = CAP biosynthetic gene promoter, mRNA_ca_ = CAP biosynthetic gene mRNA. (**C**) Encapsulates the Cap^+^ to Cap^-^ switch and maintenance of Cap^-^. Since the low UXP signal triggers Cap^+^, we require Cap^+^ to induce temporary relief to capsule production. To achieve this, we invoke a repressor triggered by Cap^+^, via direct regulation by CAP. We assume cooperativity and dimerization in the genetic regulation so as to achieve clear delineation between high and low CAP states. (**D**) Defines the turnover of mRNA and protein in the cell. Degradation of the CAP transcriptional repressor allows the Cap^-^ state to end. Thus, the time scale of protein degradation determines the length of time before the system can respond to the low UXP signal again. ø = simple molecular by-products.
(TIFF)

S6 Fig Growth-capsulation model simulations of CAP expression at high, intermediate, and low pyrimidine pathway flux. Three example outcomes of simulations of the growth-capsulation model at each of three different UXP levels (a measure of pyrimidine pathway flux) are provided. Each simulation shows how the expression of CAP changes in a single over time (h). At a high UXP level (3; **A**), such as that expected in the ancestral genotype, individual cells mainly express CAP at a basal (intermediate) level but show occasional high (Cap^+^) or low (Cap^-^) expression as indicated. At a low UXP level (0.003; **B**), such as that expected in 1B^4^ and other switcher genotypes, CAP expression typically varies stochastically between the high and low states. Stochastic switching between all three expression levels is typical at intermediate UXP level (0.3; **C**).
(TIFF)

S7 Fig Altering the model reaction rates allows the frequency of capsule switching to be tuned. Cumulative distribution plots showing simulation output for the cumulative amount of time spent with a particular level or less of CAP expression, under a variety of model parameter conditions. The simulation was run under 47 sets of conditions (obtained by sequentially increasing or decreasing by a factor of 10 each of the 23 reaction rates listed in S5 Fig, plus the original reaction conditions; see also S1 Code and S2 Code). The dark blue line is the simulation output under the original reaction conditions. Each of the lines on the plot constitutes the mean output of ten independent simulations. All sets of parameters tested generated two humped plots (the first hump is at Cap = 0) indicating two states: Cap^-^ and Cap^+^. Three reaction rate alterations resulted in Cap production in excess of 100 molecules of CAP.
(TIFF)

S1 Table Transposon mutants in which 1B^4^ colony switching is eliminated. Genotypic and phenotypic details of 157 1B^4^ transposon mutants in which the transposon insertion affects bistable capsule expression. Mutants were obtained by screening approximately 68,750 transposon mutants from 41 independent conjugations for loss of colony-level switching. Mutants are classified according to the cellular function likely to be affected by the capsulation-altering insertion. Where available, the precise genomic location of the transposon is indicated as the first base on the genomic forward strand downstream of the 3ʹ transposon terminus. For mutants of particular interest, a Cre-deletion (removing most of the transposon and thus eliminating polar effects) was obtained and analysed. See S3–S6 Tables for details of the results described in the “mRNA-seq Data Notes” column. P_ = promoter.
(XLSX)

S2 Table Analysis of the capsule polymer monosaccharide composition. Analysis of the monosaccharide composition of polymer isolated from SBW25, 1A^4^, 1B^4^, JG176ΔCre (1B^4^ *ndk* transposon mutant), and JG114ΔCre (1B^4^ *galU* transposon mutant). The analysis is split into three worksheets. The first presents the dry weight (after freeze-drying) of the polymer sample isolated from each genotype. The second lists the monosaccharide composition of each of two replicates of the polymer samples. Each measurement is presented as % w/w and is expressed as a percentage of the detected sugars (i.e., has been “normalised”). The third worksheet contains the averages, standard errors, and graphs for the analysis.
(XLSX)

S3 Table A comparison of gene expression data from SBW25 (1A^0^) and 1A^4^. Comparison showing the effects of mutations 1–8 in the evolutionary series (see Fig. 1A) on gene expression. Complete dataset available at the National Center for Biotechnology Information (NCBI) Gene Expression Omnibus (GEO) (accession number GSE48900). The table is a list of genes identified by mRNA-seq as having statistically significantly (adjusted *p*-value <0.01) different mRNA levels in genotype 1 and genotype 2 (numbered in the order listed above). Each list is split into two worksheets: (a) genotype1_higher and (b) genotype2_higher. Within each worksheet, entries are organised in order of statistical significance (lowest to highest adjusted *p*-value) and subsequently by fold change. Column A is “Locus” (the n-th gene in the SBW25 genome; note that while this correlates with *pflu* number, it is not necessarily the same); column B is “Gene Name” (assigned name or *pflu* number); column C is “Product” (protein product of the gene); column D is “EC Number” (enzyme catalogue number, where available); column E is “Colour in SBW25 Genome” (colours 1–15 each indicate a distinct functional class of gene product); columns F and G are “Genotype1 BaseMean” and “Genotype2 BaseMean,” respectively (BaseMean is the normalized mean expression level of two replicates of the relevant genotype); column H is “Fold Change” (calculated by dividing the BaseMean of each genotype as indicated); column I is “*p*-value” (calculated by assuming a binomially distributed read coverage analogous to Fisher’s exact test [51,61]); and column J is “Adjusted *p-*value” (the *p*-value adjusted for multiple testing with the Benjamini-Hochberg procedure [controls for false discovery rate] [51]).
(XLSX)

S4 Table A comparison of gene expression data from 1A^4^ and 1B^4^-Cap^-^. Comparison showing the effects of the c2020t *carB* mutation on gene expression in the Cap^-^ phenotype of 1B^4^. For the remainder of the legend, see the S3 Table legend.
(XLSX)

S5 Table A comparison of gene expression data from 1A^4^ and 1B^4^-Cap^+^. Comparison showing the effects of the c2020t *carB* mutation on gene expression in the Cap^+^ phenotype of 1B^4^. For the remainder of the legend, see the S3 Table legend.
(XLSX)

S6 Table A comparison of gene expression data from 1B^4^-Cap^-^ and 1B^4^-Cap^+^. Comparison showing the effects of capsule phenotype (+ or-) on 1B^4^ gene expression. For the remainder of the legend, see the S3 Table legend.
(XLSX)

S7 Table Intracellular concentrations of UMP, UDP, and UTP across six genotypes using LC-MS. The table contains raw and analysed data for the measurement of intracellular UMP, UDP, and UTP concentrations (nmol cfu^-1^) for five independent samples of six genotypes: SBW25, SBW25-Δ*ndk*, 1A^4^, 1B^4^, 1B^4^-Δ*ndk*, and JG176ΔCre (1B^4^ *ndk* transposon mutant). See also Fig. 4B.
(XLS)

S8 Table Intracellular concentrations of nucleotide and other components across a range of genotypes using radiolabelling. The table lists approximate intracellular concentrations (μM) of 22 small molecules (named in column B, with common abbreviations listed in column A). The 22 molecules were measured across eight genotypes (columns C–AG): SBW25, 1A^4^, 1B^4^ (under multiple conditions), Re1_4, Re1_5, JG176ΔCre (1B^4^ *ndk* transposon mutant), JG114ΔCre (1B^4^ *galU* transposon mutant), and JG74ΔCre (1B^4^ *gacA* transposon mutant). The concentration of each molecule was measured in two or three independent replicates for each genotype (samples 1, 2, and 3; worksheet titled). Individual concentrations are presented in the worksheet entitled “individual measurements,” while means and standard errors for each molecule-genotype combination are listed in the worksheet entitled “averages.” Note that UDP-glucose and UDP-galactose, both of which are building blocks for *E*. *coli* colanic acid, cannot be reliably distinguished by this method and are presented as a combined concentration (UDPG). “Undetermined” means that the concentration was unobtainable, while measurements of 0 were used to represent concentrations below the limit of detection.
(XLS)

S9 Table List of changes in model reaction rates for simulations shown in S7 Fig. The table lists each of the 47 different model reaction rates (see S5 Fig for a list of reactions) under which the simulations in S7 Fig were run. Each of the 23 listed reaction rates was sequentially increased and decreased by a factor of 10. (The 47th reaction rate is the original reaction rate used to generate Fig. 6B).
(XLS)

S1 Text Disproving genetic models of capsule switching. (DOCX)

S2 Text Extended experimental procedures. Detailed methods for the capsule counting assay, genotype constructions, and bacterial strains used.
(DOCX)

S3 Text The effect of intracellular purine levels on capsule switching. (DOCX)

S1 Video Time-lapse video following the growth of a Cap^-^ 1B^4^-CAP-GFP cell into a micro-colony. Video made from images captured every 5 min over 12 h and 30 min.
(MOV)

S2 Video Time-lapse video following the growth of a Cap^+^ 1B^4^-CAP-GFP cell into a microcolony. Video made from images captured every 10 min over 4 h.
(MOV)

## Abbreviations

ADP: adenosine diphosphate
ATP: adenosine triphosphate
BLAST: Basic Local Alignment Search Tool
BLASTP: Basic Local Alignment Search Tool protein
CAP: colanic acid-like polymer
CP: carbamoyl phosphate
CPSase: carbamoyl phosphate synthetase
CTP: cytidine triphosphate
EPS: extracellular polysaccharide
GDP: guanosine diphosphate
GFP: green fluorescent protein
KB: King’s Medium B
LB: lysogeny broth
LC-MS: liquid chromatography mass spectrometry
NDK: nucleoside diphosphate kinase
NDP: nucleoside diphosphate
NTP: nucleoside triphosphate
SOAP: short oligonucleotide alignment program
TBLASTN: translated Basic Local Alignment Search Tool nucleotides
UDP: uridine diphosphate
UMP: uridine monophosphate
UTP: uridine triphosphate
