# Supplementary figures and images for: Bistability in a Metabolic Network Underpins the De Novo Evolution of Colony Switching in Pseudomonas fluorescens
Source: PLoS Biol. 2015 Mar 12;13(3):e1002109. doi: 10.1371/journal.pbio.1002109 (PMC4357382; doi:10.1371/journal.pbio.1002109)

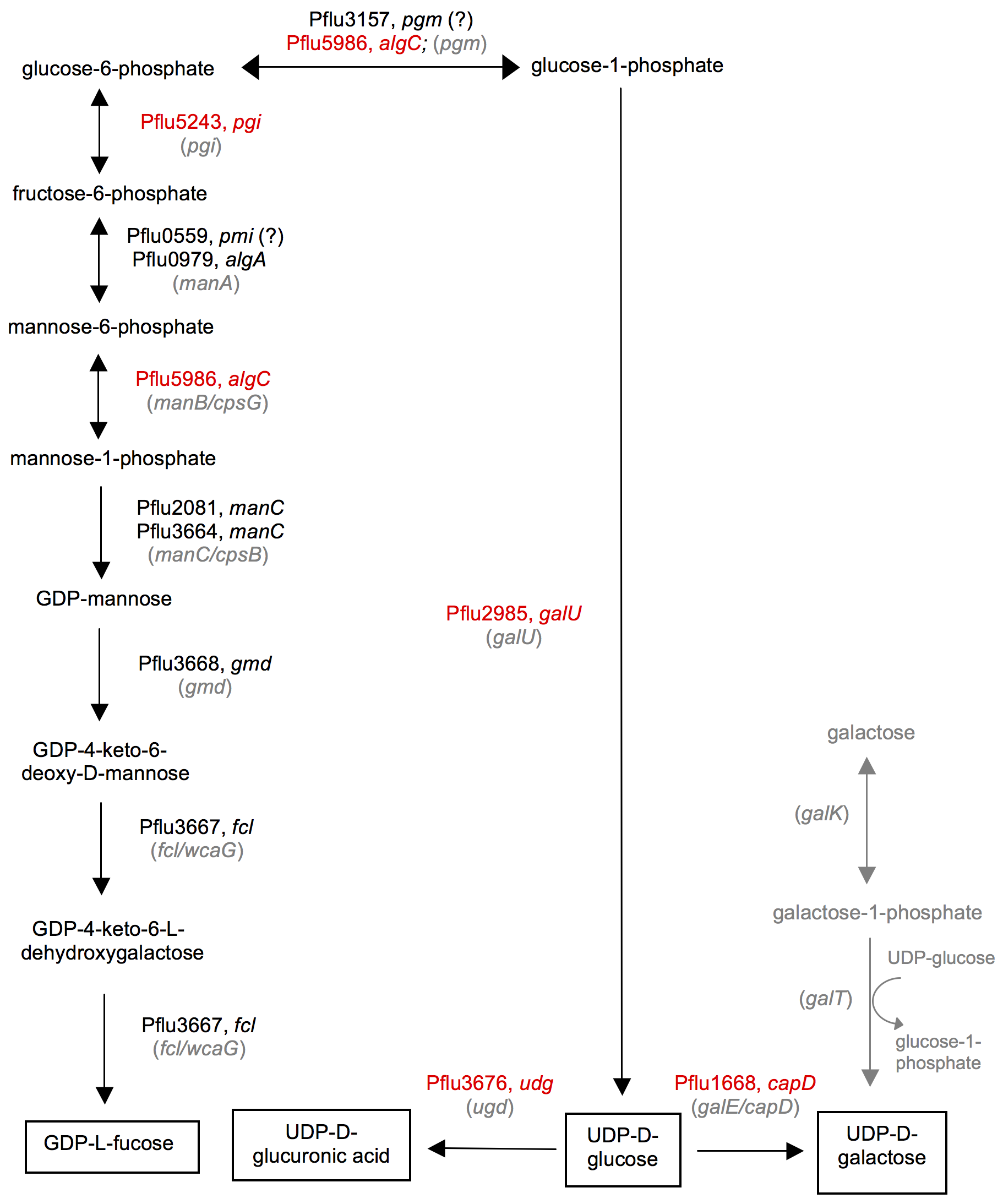

Supplement: S1 Fig — Pathways for the biosynthesis of CAP building blocks in SBW25. Genes encoding enzymes are shown by pflu number (and name where appropriate; red = recorded transposon mutant [see S1 Table and Fig. 3]). Corresponding E. coli K-12 gene names are shown in grey in parentheses [58]. The E. coli UDP-galactose biosynthetic pathway (shown in grey on right) is absent in SBW25. (TIFF) [file pbio.1002109.s010.tiff]

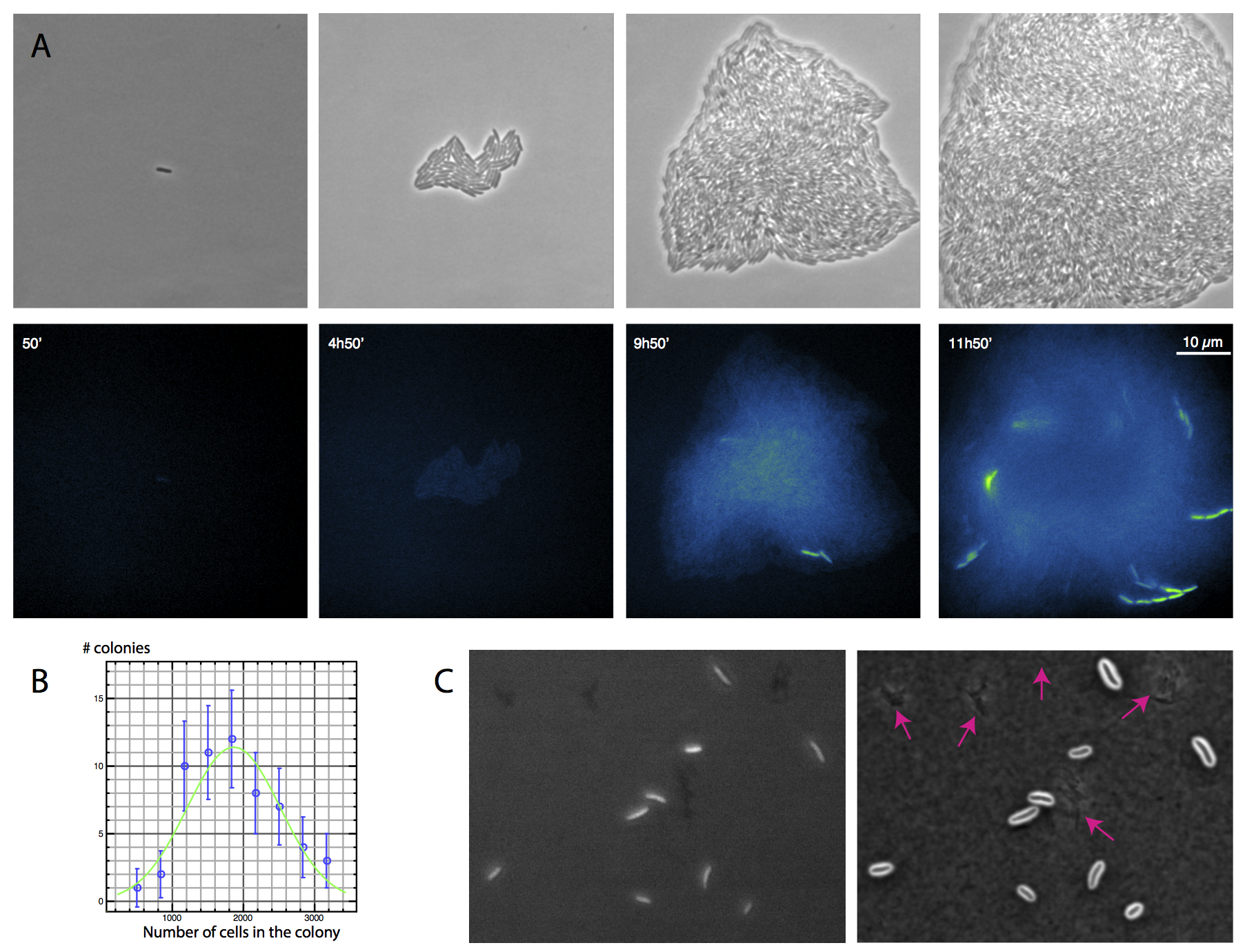

Supplement: S2 Fig — (A) Images from a time-lapse video of a single Cap- 1B4-CAP-GFP cell growing into a microcolony. At each of four time points over ~15 h, a phase-contrast image (top) and fluorescence image (bottom, Cap+ cells glow green) were captured. (B) Fit of the Gaussian distribution for the population size at which capsule switching occurs. (C) A comparison of 1B4-CAP-GFP cells under fluorescence imaging (left) and stained with India ink (right) shows that Cap+ cells reliably fluoresce, while Cap- cells (indicated by arrows) reliably do not. (TIFF) [file pbio.1002109.s011.tiff]

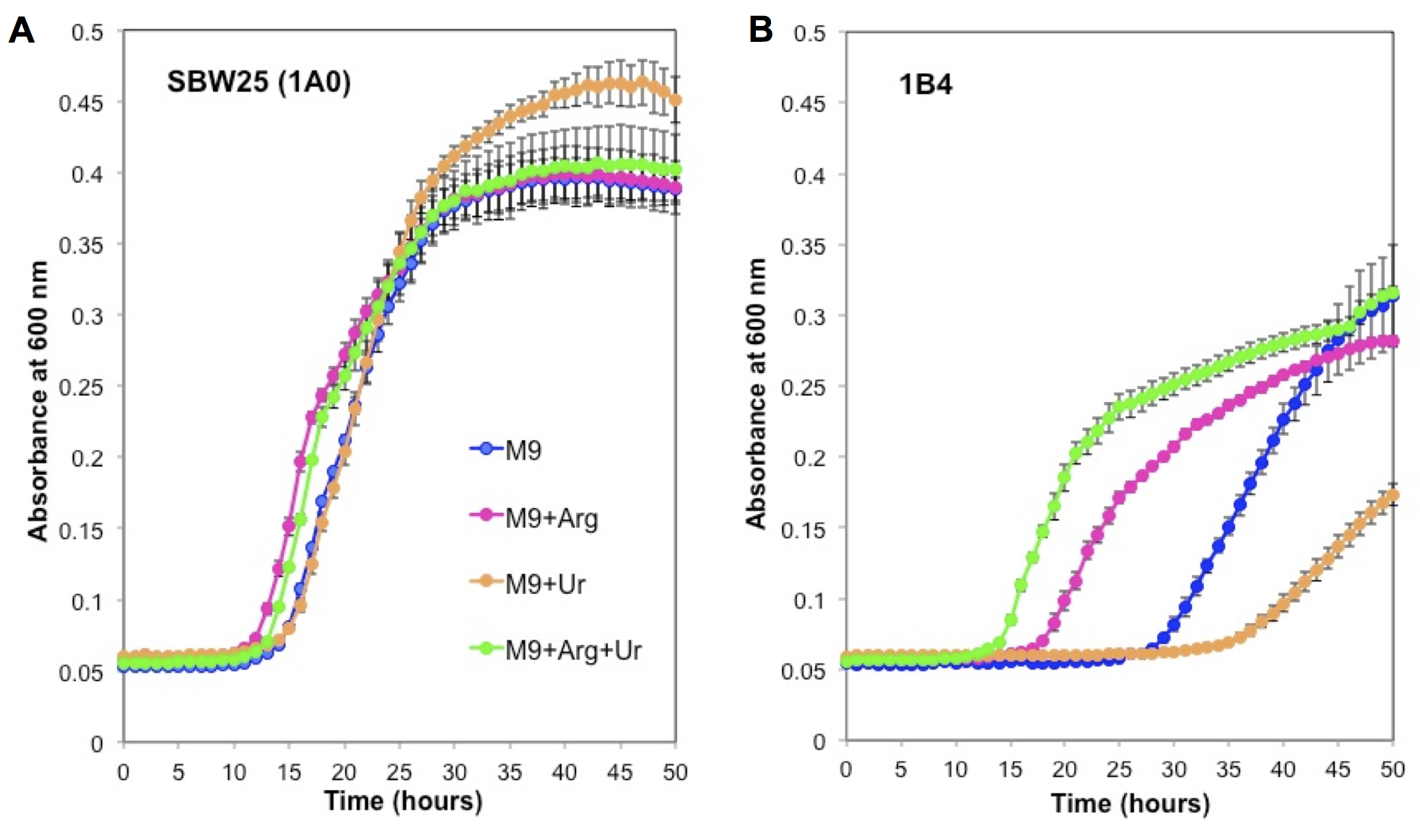

Supplement: S3 Fig — Overnight cell cultures of SBW25 (A) and 1B4 (B) were produced from glycerol stocks in shaking KB microcosms. A 1-mL aliquot of each was washed and resuspended in M9 medium to a common cell density (based on OD600 of the KB culture). 2-μL aliquots of resuspension were used to inoculate 148 μL of appropriate fresh medium in wells of a 96-well plate. Four types of media were used: M9 (blue lines on graphs), M9 + arginine (0.6 mM, pink lines), M9 + uracil (1 mM, orange lines), and M9 + arginine + uracil (green lines). Each of the eight genotype-medium combinations was replicated five times, and the 16 wells were used as media controls. The OD600 of each well was measured at 5-min intervals for 50 h, with 5 sec shaking prior to each read (using a VERSAmax plate reader). Hourly time-point data was plotted (data points are means of 5 replicates +/- 1 SE). While 1B4 grows more slowly than SBW25 in M9, 1B4 is not an auxotroph. The 1B4 growth rate deficiency is partially alleviated by addition of arginine, an effect that is increased by the addition of both arginine and uracil. The addition of uracil alone results in an even slower 1B4 growth rate than observed in M9. It is likely that this result reflects the complex regulatory systems governing CPSase biosynthesis and activity; pyrimidines have been shown to repress the transcription and activity of E. coli CPSase [59,60]. (TIFF) [file pbio.1002109.s012.tiff]

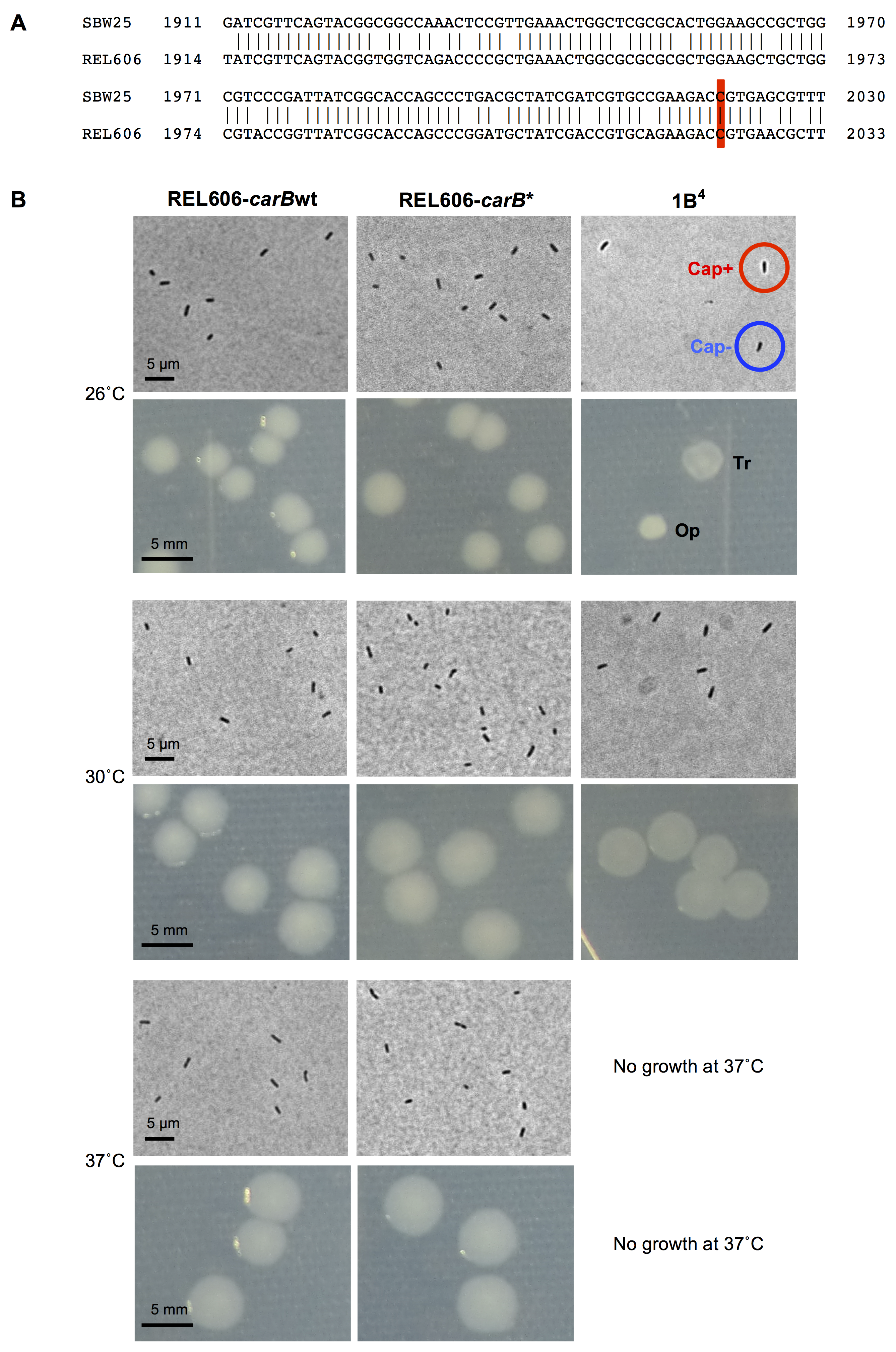

Supplement: S4 Fig — A carB mutation was constructed in E. coli B REL606. The mutation constructed was c2023t, the equivalent to the SBW25 c2020t carB mutation (A; a section of SBW25 carB aligned with that of REL606, base of interest highlighted in red). Phenotypic analysis of the constructed genotype (REL606-carB*), together with REL606-carBwt (containing wild-type carB, isolated from the same procedure as REL606-carB*) and 1B4, revealed no sign of switching at either the cellular or colony level in the constructed genotype (B). Phenotypic assays were conducted at three temperatures (26°C, 30°C, and 37°C) because CAP expression in 1B4 was found to be extremely sensitive to temperature, showing no expression over 28°C. Cells of each genotype were grown in LB medium for ~16 h and then transferred to fresh LB medium for ~24 h. Colonies were grown from LB cultures on LB agar for 24 (37°C) or 48 (30°C and 26°C) h. (TIFF) [file pbio.1002109.s013.tiff]

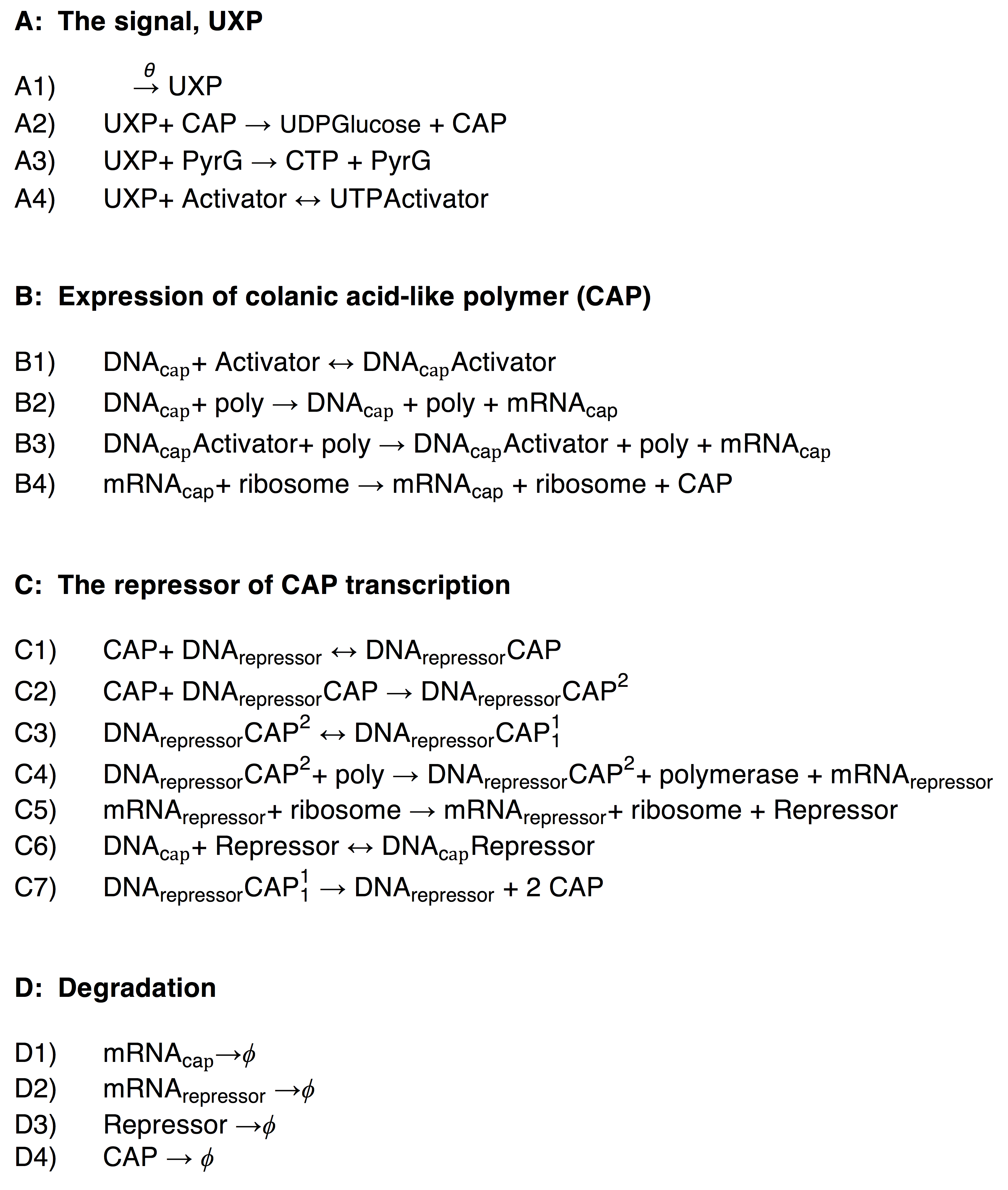

Supplement: S5 Fig — The equations describe intracellular conditions influencing the capsule switch decision (Fig. 6), demonstrating that a minimal set of components can theoretically give rise to switching. All components and interactions could be substituted so long as low UXP (signal molecule) leads to a bias in UTP utilization towards CAP biosynthesis. The system positive regulator (activator) remains constant for simplicity. (A) Describes processes affecting concentrations of the signal molecule, UXP. In the actual biological system, UXP production requires a multistep pathway; for simplicity, we model it as a constant rate (Equation A1). There are two branches for UTP utilization (Fig. 2); in the model, CAP synthesis is regulated solely by CAP (equation A2), and nucleotide synthesis is regulated solely by PyrG (Equation A3). To bias UTP utilization towards CAP under low UXP, the model assumes that UXP sequesters a transcriptional activator for the CAP biosynthetic genes (Equation A4). CA = CAP. (B) Encapsulates intracellular processes controlling the transcription of CAP biosynthetic genes. The primary purpose is to enable low UXP to signal CAP production. poly = DNA polymerase, DNAca = CAP biosynthetic gene promoter, mRNAca = CAP biosynthetic gene mRNA. (C) Encapsulates the Cap+ to Cap- switch and maintenance of Cap-. Since the low UXP signal triggers Cap+, we require Cap+ to induce temporary relief to capsule production. To achieve this, we invoke a repressor triggered by Cap+, via direct regulation by CAP. We assume cooperativity and dimerization in the genetic regulation so as to achieve clear delineation between high and low CAP states. (D) Defines the turnover of mRNA and protein in the cell. Degradation of the CAP transcriptional repressor allows the Cap- state to end. Thus, the time scale of protein degradation determines the length of time before the system can respond to the low UXP signal again. ø = simple molecular by-products. (TIFF) [file pbio.1002109.s014.tiff]

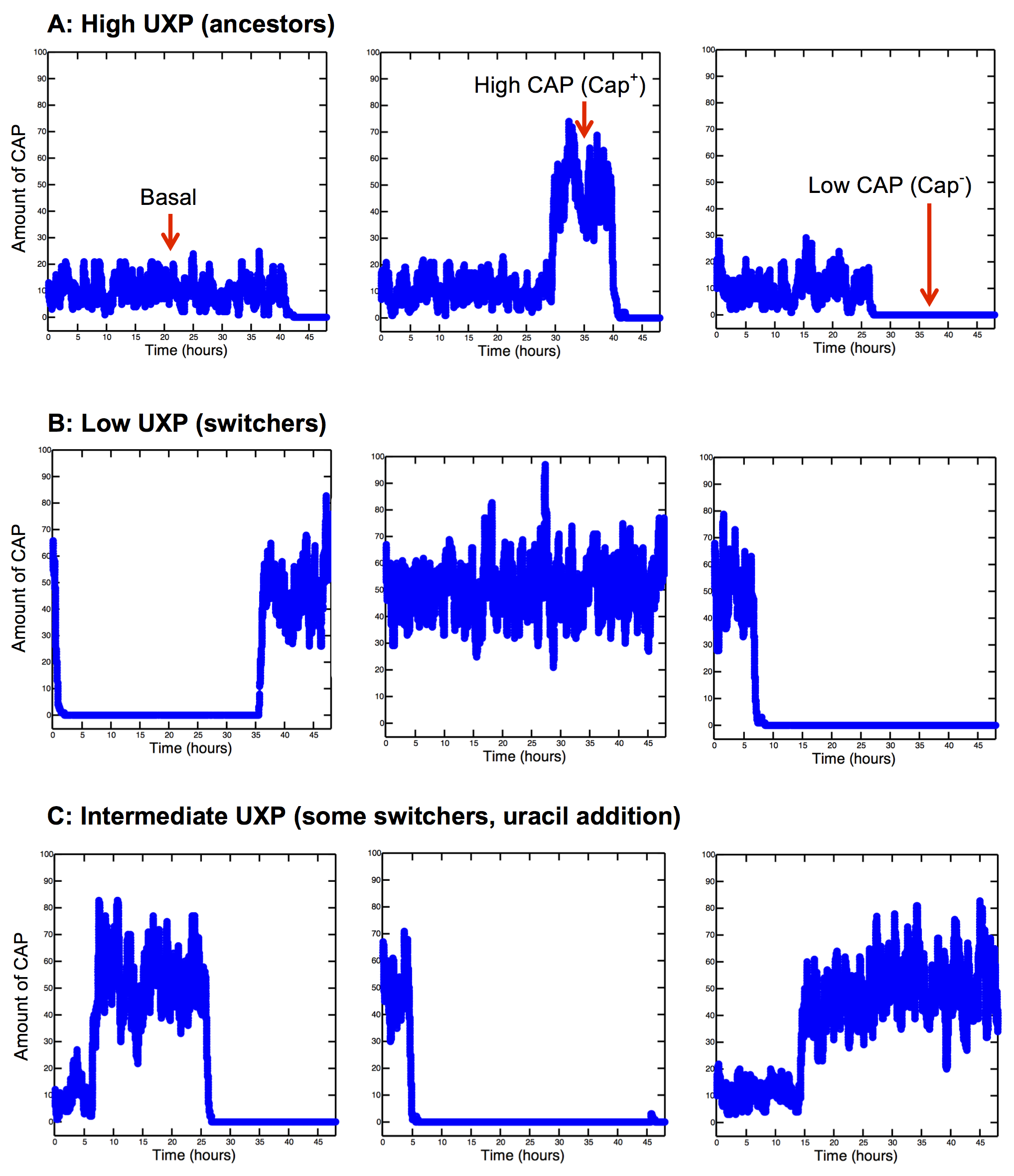

Supplement: S6 Fig — Three example outcomes of simulations of the growth-capsulation model at each of three different UXP levels (a measure of pyrimidine pathway flux) are provided. Each simulation shows how the expression of CAP changes in a single over time (h). At a high UXP level (3; A), such as that expected in the ancestral genotype, individual cells mainly express CAP at a basal (intermediate) level but show occasional high (Cap+) or low (Cap-) expression as indicated. At a low UXP level (0.003; B), such as that expected in 1B4 and other switcher genotypes, CAP expression typically varies stochastically between the high and low states. Stochastic switching between all three expression levels is typical at intermediate UXP level (0.3; C). (TIFF) [file pbio.1002109.s015.tiff]

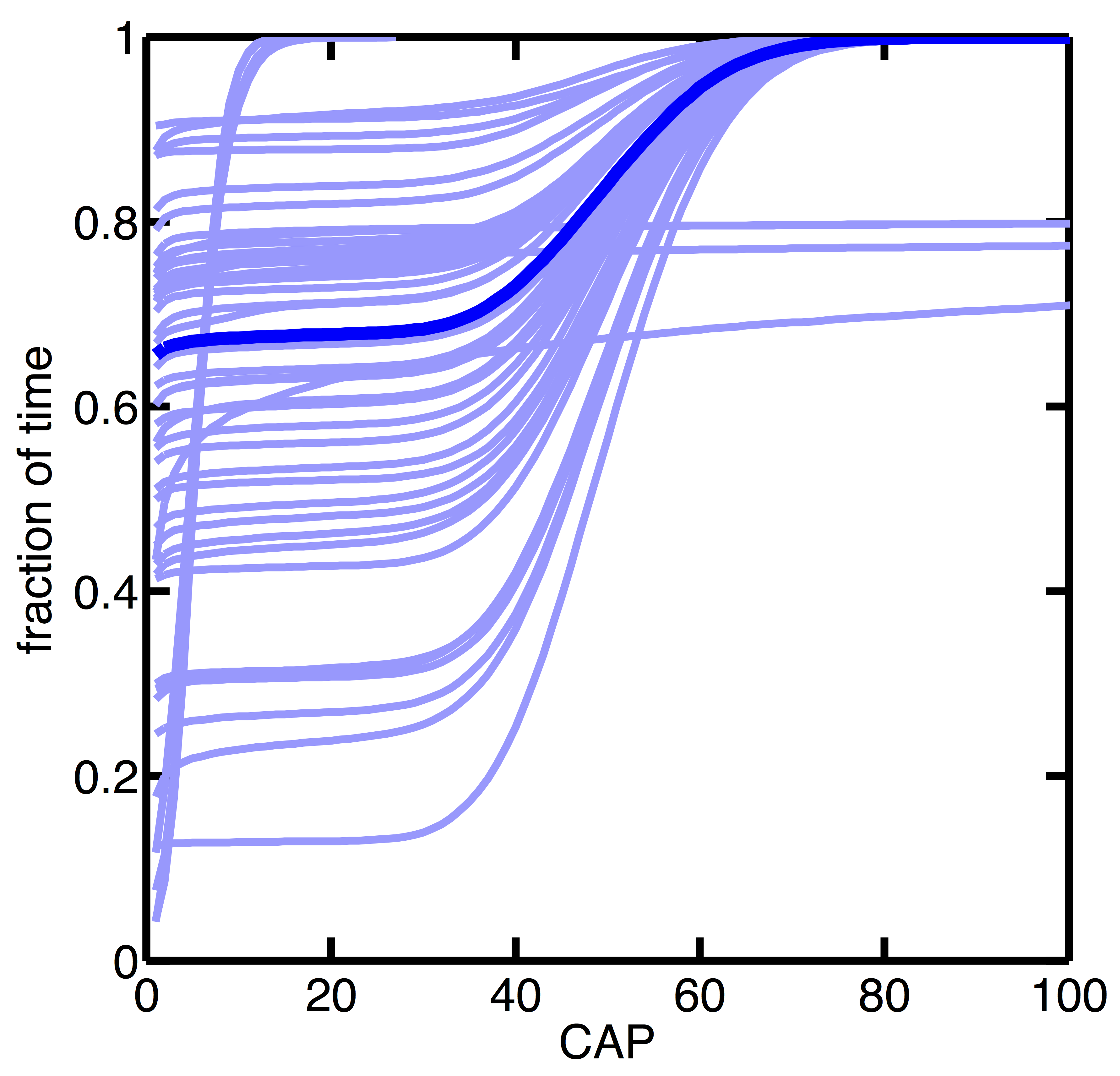

Supplement: S7 Fig — Cumulative distribution plots showing simulation output for the cumulative amount of time spent with a particular level or less of CAP expression, under a variety of model parameter conditions. The simulation was run under 47 sets of conditions (obtained by sequentially increasing or decreasing by a factor of 10 each of the 23 reaction rates listed in S5 Fig, plus the original reaction conditions; see also S1 Code and S2 Code). The dark blue line is the simulation output under the original reaction conditions. Each of the lines on the plot constitutes the mean output of ten independent simulations. All sets of parameters tested generated two humped plots (the first hump is at Cap = 0) indicating two states: Cap- and Cap+. Three reaction rate alterations resulted in Cap production in excess of 100 molecules of CAP. (TIFF) [file pbio.1002109.s016.tiff]
