# Supplementary material for: Bistability in a Metabolic Network Underpins the De Novo Evolution of Colony Switching in Pseudomonas fluorescens
Source: PLoS Biol. 2015 Mar 12;13(3):e1002109. doi: 10.1371/journal.pbio.1002109 (PMC4357382; doi:10.1371/journal.pbio.1002109)
Supplement: S1 Text — (DOCX) [file pbio.1002109.s026.docx]

**1 High frequency gain and loss of c2020t *carB* does not cause switching**

Given that 1B^4^ capsule bistability is caused by the c2020t *carB* mutation [1], rapid gain and loss of the mutation is the simplest mechanistic explanation. Analysis of the DNA sequence surrounding the c2020t mutation failed to identify any repetitive tracts that might be prone the type prone to rapid mutational change [2]. Nonetheless, an overnight culture of 1B^4^ was separated by centrifugation into Cap^+^ and Cap^-^ fractions, and the relevant region of *carB* sequenced. The c2020t *carB* mutation was present in both fractions, showing that capsule switching is not caused by gain and loss of the *carB* mutation. This indicates that the mutation must establish a series of genetic and/or epigenetic events that culminate in phenotypic bifurcation of the population.

**2 Amplification of *carB* is not responsible for switching**

We considered the possibility that a deficit of one or more pyrimidine biosynthetic pathway intermediates (due to the *carB* mutation) might allow selection to amplify the *carB* locus in a manner akin to amplification of the partially defective *lacIZ* allele in *E. coli* grown on lactose [3]. According to this model, amplification of *carB* would increase the amount of the defective carbamoylphosphate (CP) enzyme to the point of over supply leading to expression of the Cap^+^ state. The resulting population would then experience relaxed selection for maintenance of the amplified locus resulting in a reduction in copy number, a concomitant reduction in CP, and a switch to the Cap^-^ state. Cells returned to the CP deficient state would stand to continue to cycle between high and low states (and thus Cap^+^ and Cap^-^ cells) as a consequence of expansion and contraction of the *carB* locus. The model depends on the *carB* mutation reducing CarAB function (and thus CP levels), and predicts changes in *carB* copy number to underpin capsule switching.

Compared to the ancestral genotype (1A^0^), and the immediate precursor genotype (1A^4^), 1B^4^ grows slowly on minimal medium. The defect is alleviated by addition of uracil and arginine (S3 Fig.). The *carB* mutation thus appears to reduce gene function, thus satisfying conditions that would favor selective amplification of the locus. To test the prediction that changes in *carB* copy number underpin switching, the *carB* locus was deleted from 1B^4^. Removal of *carB* eliminates the possibility that the locus can be targeted by selection: the mutant should be incapable of switching capsulation state. In addition, because amplification of *carB* is likely to depend on homologous recombination, *recA* was deleted from 1B^4^. Both *carB* and *recA* mutants showed evidence of opaque and translucent colonies (and Cap^+^/Cap^-^ cells) on agar plates. Microscopy was used to determine the proportion of capsulated 1B^4^-∆*carB* and 1B^4^-∆*recA* cells relative to 1B^4^. No significant difference was observed in 1B^4^ compared to 1B^4^-∆*carB* (*t*-test *p*=0.307) or 1B^4^-∆*recA* (*t*-test *p*=0.847). Finally, high-throughput genome sequencing of 1B^4^ showed no evidence of amplification (or reduction) at *carB*, or elsewhere in the genome. Further, no genetic differences were detected between Cap^+^ and Cap^-^ cells. Together these data led to rejection of the amplification model.

**References**

**References**

1. Beaumont HJE, Gallie J, Kost C, Ferguson GC, Rainey PB (2009) Experimental evolution of bet hedging. Nature 462: 90-94.

2. Moxon ER, Rainey PB, Nowak MA, Lenski RE (1994) Adaptive evolution of highly mutable loci in pathogenic bacteria. Curr Biol 4: 24-33.

3. Andersson DI, Slechta ES, Roth JR (1998) Evidence that gene amplification underlies adaptive mutability of the bacterial *lac* operon. Science 282: 1133-1135.
