# Supplementary material for: Bistability in a Metabolic Network Underpins the De Novo Evolution of Colony Switching in Pseudomonas fluorescens
Source: PLoS Biol. 2015 Mar 12;13(3):e1002109. doi: 10.1371/journal.pbio.1002109 (PMC4357382; doi:10.1371/journal.pbio.1002109)
Supplement: S2 Text — Detailed methods for the capsule counting assay, genotype constructions, and bacterial strains used. (DOCX) [file pbio.1002109.s027.docx]

**1 Capsule Counting Assay**

Quantitative measures of Cap^+^ proportions were obtained in populations of interest, according to the following protocol. For each genotype of interest three or five replicate KB microcosms were inoculated from -80˚C glycerol stocks. Microcosm lids were loosened, and the microcosms incubated for 24 hours (160-180 rpm). Following vortexing for 1 minute, 6 µL aliquots of cells from each pre-culture were used to inoculate fresh KB microcosms. After incubation for a further 24 hours, each microcosm was vortexed for 1 minute.

Cells were diluted 1:5 in sterile dH_2_O, mixed thoroughly with India ink, and allowed to incubate at room temperature for approximately 1 minute. Microscope slides were prepared by dropping 6 µl of the mixture onto the slide and covering with a glass cover slip. Care was taken to avoid the formation of air bubbles, as these cause non-homogeneous cell distributions. Slides were left at room temperature for approximately 2 minutes to reduce Cap^-^ cell motility. Then, each slide was placed under the light microscope, viewed under phase contrast x40 or x63 magnification and microscopic images recorded for later analysis.

Individual cells recorded in the microscopic images were manually assayed for capsule expression, with the aid of a computer programme. For each replicate, the state of capsule expression (ON or OFF) was recorded for 500 cells; a maximum of 100 cells were recorded *per* image to avoid bias from cells accumulating in particular areas of the microscope slide. Finally, average and standard error values were determined for each genotype, and statistical tests performed to detect differences between capsulation proportions in genotypes of interest.

**2 Genotype Constructions**

*2.1 Gene deletions*

Four large-scale deletions were made in the 1B^4^ background (the fourth, *ndk*, was also made in the SBW25 background; see Table below for primer details):

1. The *recA* gene (which covers bases 1318115-1319173). Bases 1318086-1319155 inclusive were replaced with a 23 bp sequence.
2. All of *carB* (which covers bases 5781306-5778085) except for the final 34 bases, which are also part of *greA*. Bases 5778119-5781318 inclusive were replaced with a 23 bp sequence.
3. The CAP structural gene locus, *wcaJ-wzb* (which covers bases 4050846-4074200). Bases 4050675-4074194 inclusive were replaced with a 23 bp sequence.
4. The *ndk* gene (which covers bases 5562180-5561755). Bases 5560946-5562929 inclusive were deleted (cleanly, with no replacement sequence).

For each, a deletion fragment was created by SOE-PCR [1] and ligated into the *Pseudomonas* suicide vector, pUIC3 [2]. The vector construct was inserted into chemically competent *E. coli* DH5α-λ*pir* cells and transferred to 1B^4^ *via* tri-parental conjugation (helper strain was *E. coli* carrying pRK2013, which contains the *tra* and *mob* genes necessary for conjugation). The deletion fragment and the target wild type chromosomal segment were then exchanged *via* two-step allelic exchange [3]. Double cross over mutants were purified and then checked for Tc sensitivity and the formation of white colonies on LB+X-gal (*i.e.*, loss of pUIC3), and for the incorporation of the deletion fragment by PCR and sequencing.

| **Primer Name** | **Sequence (5**'**🡪3**'**)^1^** | **Use** |
| --- | --- | --- |
| RecAKO-1 | gaagatctCAATTGGCTGCCGAACTGGG | Deletion of *recA* |
| RecAKO-2b | cagcatgcggatccgttgacggaGGCGGCCAACACCTGTATAAG | Deletion of *recA* |
| RecAKO-3 | tccgtcaacggatccgcatgctgGCTGACACTGACATCTGAAGC | Deletion of *recA* |
| RecAKO-4 | gaagatctCAAAGCTCCCTTGCCACAGC | Deletion of *recA* |
| CarB1 | gaagatctGTGCGGGCTGTATAACCAG | Deletion of *carB* |
| CarB2 | cagcatgcggatccgttgacggaCTTGCAGGACCTACACGCAG | Deletion of *carB* |
| CarB5 | tccgtcaacggatccgcatgctgCAAATCCGCAGGTCAGTCG | Deletion of *carB* |
| CarB6 | gaagatctGATGTCGCTGTCCGATTACC | Deletion of *carB* |
| wcaJKO-1 | gaagatctcgcagacgctgaacacctg | Deletion of *wcaJ-wzb* |
| wcaJKO-2 | cagcatgcggatccgttgacggacgtgtgttgataggcagggtg | Deletion of *wcaJ-wzb* |
| wcaJKO-5 | tccgtcaacggatccgcatgctgGAACAAGGCAACCTCCCACG | Deletion of *wcaJ-wzb* |
| wcaJKO-6 | gaagatctCTTTCTCAGCGCGTAACTGCG | Deletion of *wcaJ-wzb* |
| oPR54 | gtagatctACCCAAGGTCGCCATATAACG | Deletion of *ndk* |
| oPR55 | AGTTACACCATGGCTTAAGCATCGGCTTAAAGAGTGAG | Deletion of *ndk* |
| oPR56 | TAAGCCGATGCTTAAGCCATGGTGTAACTCCAGAAAC | Deletion of *ndk* |
| oPR57 | gtagatctGTCTGTCGCAAGTGACCGATG | Deletion of *ndk* |

^1^underline denotes *Bgl*II restriction site. Capital letters denote homology to the SBW25 genome sequence, and small letters denote primer sequence that does not match the SBW25 genome sequence.

*2.2 Mutation construction in* E. coli *B REL606*

To investigate whether the *carB* mutation causes obvious phenotypic switching in an *E. coli* background, the SBW25 c2020t *carB* mutation (causing amino acid change R674C) was constructed in the *E. coli* B strain, REL606 (see Table below for primers used). According to gene alignments (see S4 Fig.) the equivalent mutation in REL606 is c2023t (causing amino acid change R675C). The mutation was constructed using the scar-free, pKOV-mediated two-step allelic exchange protocol [4]. The resulting genotype, REL606-*carB**, was confirmed to contain the c2023t *carB* mutation by PCR amplification and Sanger sequencing of the relevant region. A strain containing wild type *carB*, REL606-*carB*wt, was also isolated from the exchange procedure. This strain showed no differences to REL606-*carB** in any phenotypic tests performed (S4 Fig.).

| **Primer Name** | **Sequence (5**'**🡪3**'**)^1^** | **Use** |
| --- | --- | --- |
| carB_c2023t_1 | gaagatctCTTTGCCGATGCGCGTCTG | Construction of c2023t in REL606 |
| carB_c2023t_2 | GGAAGCGTTCACAGTCTTCTGCAC | Construction of c2023t in REL606 |
| carB_c2023t_3 | GTGCAGAAGACTGTGAACGCTTCC | Construction of c2023t in REL606 |
| carB_c2023t_4 | gaagatctCAGCGATTTGCCAGCCATCAC | Construction of c2023t in REL606 |

^1^underline denotes *Bgl*II restriction site. Highlighted in green is the mutated base. Capital letters denote homology to the REL606 genome sequence, and small letters denote primer sequence that does not match REL606 (and are not present in the final construct).

*2.3 Transcriptional* lacZ *fusion*

To investigate whether CAP expression is controlled at the transcriptional level, a 1B^4^ chromosomal transcriptional fusion was constructed between *lacZ* and the CAP structural gene *wcaJ*. The *wcaJ* gene is a good candidate for detecting difference in expression between Cap^-^ and Cap^+^ cells because (a) it is the first gene of the CAP structural locus, (b) the organisation of the SBW25 CAP structural locus (Fig. 3) suggests that *wcaJ* is transcribed alone, meaning that the insertion should have limited effects on downstream gene expression, and (c) *wcaJ* expression is absolutely required for capsule expression; 1B^4^ transposon mutants with insertions in *wcaJ* are completely incapable of capsule expression (S1 Table).

The transcriptional *lacZ* fusion was made by amplifying *wcaJ* (covers bases 4050846-4052261; the fusion fragment consisted of bases 4050809-4052265; see Table below for primers) and inserted into the *Pseudomonas* suicide vector pUIC3 [2] directly upstream of promoterless *lacZY*. The constructed vector was inserted into chemically competent *E. coli* DH5α-λ*pir* cells and transferred to 1B^4^ *via* tri-parental conjugation (the helper strain was *E. coli* carrying pRK2013, which contains the *tra* and *mob* genes necessary for conjugation). Transconjugants were screened for types that had incorporated the vector construct into the chromosome (at *wcaJ* *via* homologous recombination) by plating on LB+Tc(25 µg mL^-1^)+NF. NF inhibits *E. coli* growth and so selects against the donor and helper strains, while tetracycline inhibits growth of the recipient. Successful transconjugants (NF^R^, Tc^R^) were purified. A complete, functional copy of *wcaJ* remained in the final genome (a requirement for realisation of the Cap^+^ phenotype, see S1 Table).

| **Primer Name** | **Sequence (5**'**🡪3**'**)^1^** | **Use** |
| --- | --- | --- |
| WcaJ-lacZf | gaagatctGTATTGCGCCGCGTGATC | *CAP*-*lacZ* transcriptional fusion |
| WcaJ-lacZr | gaagatctGCGCTCAGTAGATATCCTTGG | *CAP*-*lacZ* transcriptional fusion |

^1^underline denotes *Bgl*II restriction site. Capital letters denote homology to the SBW25 genome sequence, and small letters denote primer sequence that does not match the SBW25 genome sequence.

*2.4 1B^4^-CAP-GFP, the GFP expression strain*

First, the promoter of CAP biosynthetic locus gene *pflu3655* was amplified by PCR from SBW25 genomic DNA (4047744-4048551; primers oPR37/oPR38, see below). The *gfpmut3* gene was independently amplified from the miniTn7(Gm)P_rrnB P1_*gfp*-a plasmid [5] using primers oPR39/oPR40 (see below). PCR products were checked by electrophoresis, purified and an equal quantity of each used as template for SOE-PCR [1] with primers oPR37/oPR40. The promoter_*pflu3655-gfpmut3* fusion product was ligated into pGEMT-easy (Promega) and the sequence of the insert confirmed by Sanger sequencing. The sequence-confirmed fusion fragment was inserted as a *Spe*I restriction fragment into suicide plasmid pUC18R6K-miniTn7T-Gm [6], and the vector+insert was introduced into 1B^4^ by conjugation (with helper plasmids pRK2013 and pUX-BF13). Insertion at the desired site (25 bp downstream of the *glmS* stop codon; insertion directly downstream of base 6697558) was checked by PCR and Sanger sequencing.

| **Primer Name** | **Sequence (5**'**🡪3**'**)^1^** | **Target** |
| --- | --- | --- |
| oPR37 | cagtactagtCGTTTCTCGACAGCCTGGTG | Promoter *pflu3655* (forward, *Spe*I) |
| oPR38 | tctcctttacgcatAGTCCGTGCAATAGCGAGGA | Promoter *pflu3655* (reverse) |
| oPR39 | ctattgcacggactATGCGTAAAGGAGAAGAACTT | Upstream of *gfp* (forward) |
| oPR30 | cagtactagtTATTTGTATAGTTCATCCATGC | Downstream of *gfp* (reverse, *Spe*I) |

^1^underline denotes *Spe*I restriction site. Capital letters denote homology to the SBW25 genome sequence, and small letters denote primer sequence that does not match the SBW25 genome sequence.

1B^4^-CAP-GFP was constructed with the aim of differentially marking 1B^4^-Cap^+^ and 1B^4^-Cap^-^ cells within a population; our expectation was that Cap^+^ cells (CAP ON) would express GFP and therefore be fluorescent (under the correct microscopy conditions), while Cap^-^ cells (CAP OFF) would not express GFP and thus would not fluoresce. Microscopic analysis of individual cells demonstrated a 100% correlation between capsulation and GFP expression (see S2 Fig.).

*2.5 Over-expression genotypes*

Constructing the over-expression genotypes involved PCR-amplifying the complete coding regions of *carB* (bases 5781306-5778085 inclusive), *pyrH* (1408153-1408896), *ndk* (5562180-5561755), *galU* (3252521-3253360) and *pyrG* (1427223-1428854). PCR primers were designed so that the expression fragment consisted precisely of the 5'🡪3' coding sequence of each gene (including the stop codon), flanked by a 5' *Nde*I and a 3' *BamH*I restriction site (see Table below for primer sequences). Sequence-confirmed PCR products were uni-directionally ligated into the doubly digested *Pseudomonas* expression vector, pSX [7]. Each vector construct was used to transform three biological replicates of each of 1B^4^, Re1_4 and SBW25 chemically competent cells [8]. Purified transformants were used for over-expression assays. It should be noted that pSX carries an IPTG-inducible promoter. However, in a preliminary experiment, the addition of 0.1 mM IPTG was found to have no (further) effect on capsulation levels, and so IPTG was not used during the main experiments.

| **Primer Name** | **Sequence (5**'**🡪3**'**)^1^** | **Use** |
| --- | --- | --- |
| CarBOE-f | ggaattccatATGccaaaacgtacagacataaaaagc | Overexpression of *carB* |
| CarBOE-r | cgggatccTCATGCCTTCAATCCTGCGTG | Overexpression of *carB* |
| PyrHOE-f | ggaattccatATGGCTCAGCAGGGCAGTGGTTATC | Overexpression of *pyrH* |
| PyrHOE-f | cgggatccTCATTGTTGGCCTTCCTCGATCAG | Overexpression of *pyrH* |
| NdkOE-f | ggaattccatATGgctgttcaacgtactttctccatcatc | Overexpression of *ndk* |
| NdkOE-r | cgggatccTTAGCGAGCGGTTACTTCAGTAGC | Overexpression of *ndk* |
| GalUOE-f | ggaattccatATGatcaagaaatgcttgttcccagcag | Overexpression of *galU* |
| GalUOE-r | cgggatccTCAGTAAGCCTTGCCAGTCTTGTAG | Overexpression of *galU* |
| PyrGOE-f | ggaattccatATGacgcgctacatattcgtcacg | Overexpression of *pyrG* |
| PyrGOE-r | cgggatccTTACGCCTTCTTCTGATGTTGCGTC | Overexpression of *pyrG* |

^1^underline denotes *Nde*I (CATATG) or *BamH*I (GGATCC) restriction sites. Capital letters denote homology to the SBW25 genome sequence, and small letters denote primer sequence that does not match the SBW25 genome sequence.

**3 Bacterial strains used**

| **Name** | **Description** | **Reference** |
| --- | --- | --- |
| SBW25 | Original, wild type strain; also known as 1A^0^ (below). | [9] |
| *Strains in the line 1 evolutionary series* | | |
| 1A^0^ | Original, wild type strain; also known as SBW25 (above). This name applies to the line 1 evolutionary series. | [9] |
| 1B^0^ | Contains mutation 1 (*mwsR* g2778a, causing amino acid change M926I). | [10] |
| 1A^1^ | Contains mutation 1 and 2 (*mwsR* g2383a, causing amino acid change E795K). | [10] |
| 1B^1^ | Contains mutation 1, 2 and 3 (*awsX* ∆229-261, causing the in-frame deletion of amino acids 77-87). | [10] |
| 1A^2^ | Contains mutation 1-3 and 4 (*awsR* a1141c, causing amino acid change T381S). | [10] |
| 1B^2^ | Contains mutation 1-4 and 5 (*wspF* 157insG, causing a frame shift and altering amino acid sequence from M52 (236 new residues until stop codon reached). | [10] |
| 1A^3^ | Contains mutation 1-5 and 6 (*wssA* 164insA, causing a frame shift and altering amino acid sequence from S54 (28 new residues until stop codon reached). | [10] |
| 1B^3^ | Contains mutation 1-6 and 7 (*mwsR* c3094g, causing amino acid change R1032G). | [10] |
| 1A^4^ | Immediate ancestor of the switcher genotype; contains mutation 1-7 and 8 (*mwsR* ∆c2553, causing a frame shift and altering amino acid sequence from D851 (2 new residues until stop codon reached). | [10] |
| 1B^4^ | Switcher genotype; contains mutation 1-8 and 9 (*carB* c2020t, causing amino acid change R674C). | [10] |
| *Switcher strains independently isolated from 1A^4^ (all containing mutations 1-8 and 1 novel mutation)* | | |
| Re1_5 | Switch-causing mutation is *carB* c836t causing amino acid change T279I. | [10] |
| Re2 | Switch-causing mutation is *carB* a2477g causing amino acid change N826S. | [10] |
| Re1_2 | Switch-causing mutation is *carB* c431t causing amino acid change P144L. | [10] |
| Re12 | Switch-causing mutation is *carB* g695a causing amino acid change C232Y. | [10] |
| Re1_8 | Switch-causing mutation is *carB* c2020t causing amino acid change R674C; isogenic to 1B^4^. | [10] |
| Re1_4 | Switch-causing mutation is *pyrH* c331t causing amino acid change R123C. | [10] |
| *Deletions and fusions* | | |
| 1B^4^-∆*carB* | 1B^4^ with a complete, scar-free deletion of the *carB* gene. | This study |
| 1B^4^-∆*recA* | 1B^4^ with a complete, scar-free deletion of the *recA* gene. | This study |
| 1B^4^-∆*wcaJ-wzb* | 1B^4^ with a complete, scar-free deletion of the CAP biosynthetic locus (*wcaJ-wzb*). | This study |
| 1B^4^-∆*ndk* | 1B^4^ with a complete, scar-free deletion of *ndk.* | This study |
| SBW25-∆*ndk* | SBW25 with a complete, scar-free deletion of *ndk.* | This study |
| 1B^4^-*wcaJ-lacZ* | 1B^4^ with a *wcaJ*-*lacZ* transcriptional fusion (*wcaJ* inserted into *Bgl*II site of pUIC3) recombined into the 1B^4^ chromosome at *wcaJ*; Tc^R^, Amp^R^, *lacZ*. | This study |
| 1B^4^-CAP-GFP | 1B^4^ with a fluorescent marker for capsule expression; 1B^4^ containing a “promoter-*gfp-*vector” insert at the *glmS* locus. The insert is the promoter of *pflu3655* fused to *gfpmut3*, inserted into pUC18R6K-miniTn7T-Gm. Gm^R^. | This study |
| *Transposon mutant strains discussed in main manuscript* | | |
| JG176 | 1B^4^ transposon mutant with insertion in *ndk* (at genome base 5562166). More Cap^+^ than 1B^4^. Km^R^. | This study |
| JG176∆Cre | Cre-deletion of JG176. More Cap^+^ than 1B^4^. Km^S^. | This study |
| JG114∆Cre | Cre-deletion of JG114, a 1B^4^ transposon mutant with insertion in *galU* (at genome base 3252712). Fewer Cap^+^ than 1B^4^. Km^S^. | This study |
| *Construction of the* carB *mutation in* E. coli | | |
| REL606-*carB*wt | *E. coli* B REL606 strain with a wild type *carB* gene; isolated from the same procedure as REL606-*carB** | This study |
| REL606-*carB** | *E. coli* B REL606 strain containing the c2023t mutation in the *carB* gene; isolated from the same procedure as REL606-*carB*wt | This study |
| *Over-expression strains* | | |
| 1B^4^+pSX | 1B^4^ containing empty pSX vector. Gm^R^, Amp^R^. | This study |
| 1B^4^+pSX_*carB* | 1B^4^ containing pSX vector with *carB* ligated into MCS. Gm^R^, Amp^R^. | This study |
| 1B^4^+pSX_*pyrH* | 1B^4^ containing pSX vector with *pyrH* ligated into the MCS. Gm^R^, Amp^R^. | This study |
| 1B^4^+pSX*_ndk* | 1B^4^ containing pSX vector with *ndk* ligated into the MCS. Gm^R^, Amp^R^. | This study |
| 1B^4^+pSX_*galU* | 1B^4^ containing pSX vector with *galU* ligated into the MCS. Gm^R^, Amp^R^. | This study |
| 1B^4^+pSX_*pyrG* | 1B^4^ containing pSX vector with *pyrG* ligated into the MCS. Gm^R^, Amp^R^. | This study |
| SBW25+pSX_*carB* | SBW25 containing empty pSX vector. Gm^R^, Amp^R^. | This study |
| SBW25+pSX_*pyrH* | SBW25 containing pSX vector with *carB* ligated into the MCS. Gm^R^, Amp^R^. | This study |
| SBW25+pSX*_ndk* | SBW25 containing pSX vector with *pyrH* ligated into the MCS. Gm^R^, Amp^R^. | This study |
| SBW25+pSX_*galU* | SBW25 containing pSX vector with *ndk* ligated into the MCS. Gm^R^, Amp^R^. | This study |
| SBW25+pSX_*pyrG* | SBW25 containing pSX vector with *galU* ligated into the MCS. Gm^R^, Amp^R^. | This study |
| *Escherichia coli strains used during molecular genetics* | | |
| *E. coli* DH5α-λ*pir* | supE44, ∆lacU169, *hsdR17*, *recA1*, *endA1*, *gyrA96*, thi- 1, *relA1*,λ*pir* | Invitrogen |
| *E. coli* TOP10 | F', *mcrA*, Δ(mrr-hsdRMS-mcrBC), Φ80lacZΔM15, ΔlacX74, deoR, *recA1*, *araD139*, Δ(ara-leu)7697, *galU*, *galK,* *rpsL*, Str^R^, *endA1*, *nupG* | Invitrogen |

**References**

1. Ho SN, Hunt HD, Horton RM, Pullen JK, Pease LR (1989) Site-directed mutagenesis by overlap extension using the polymerase chain reaction. Gene 77: 51-59.

2. Rainey PB (1999) Adaptation of *Pseudomonas fluorescens* to the plant rhizosphere. Environ Microbiol 1: 243-257.

3. Kitten T, Kinscherf TG, McEvoy JL, Willis DK (1998) A newly identified regulator is required for virulence in *Pseudomonas syringae*. Mol Microbiol 28: 917-929.

4. Lindsey H, Gallie J, Taylor S, Kerr B (2013) Evolutionary rescue from extinction is contingent on a lower rate of environmental change. Nature 494: 463-466.

5. Lambertsen L, Sternberg C, Molin S (2004) Mini-Tn7 transposons for site-specific tagging of bacteria with fluorescent proteins. Environ Microbiol 6: 726-732.

6. Choi KH, Gaynor JB, White KG, Lopez C, Bosio CM, et al. (2005) A Tn7-based broad-range bacterial cloning and expression system. Nat Methods 2: 443-448.

7. Owen JG, Ackerley DF (2011) Characterization of pyoverdine and achromobactin in *Pseudomonas syringae* pv. Phaseolicola 1448a BMC Microbiol 11: 218.

8. Chuanchuen R, Narasaki CT, Schweizer HP (2002) Benchtop and microcentrifuge preparation of *Pseudomonas aeruginosa* competent cells. Biotechniques 33: 760-763.

9. Rainey PB, Bailey MJ (1996) Physical and genetic map of the *Pseudomonas fluorescens* SBW25 chromosome. Mol Microbiol 19: 521-533.

10. Beaumont HJE, Gallie J, Kost C, Ferguson GC, Rainey PB (2009) Experimental evolution of bet hedging. Nature 462: 90-94.
