# Supplementary material for: Bistability in a Metabolic Network Underpins the De Novo Evolution of Colony Switching in Pseudomonas fluorescens
Source: PLoS Biol. 2015 Mar 12;13(3):e1002109. doi: 10.1371/journal.pbio.1002109 (PMC4357382; doi:10.1371/journal.pbio.1002109)
Supplement: S3 Text — (DOCX) [file pbio.1002109.s028.docx]

**The Effect of Intracellular Purine Levels on Capsule Switching**

Given that the balance between intracellular pyrimidine and purine pools is tightly regulated [1], it seemed plausible that capsule switching in 1B^4^ could result from an imbalance in pyrimidine and purine pools, rather than a reduction in pyrimidines *per se*. Thus, a further experiment was performed in which the effect of adding guanine hydrochloride (a purine) on capsule switching was examined. Growth of 1B^4^ in the presence of 1.5 mM guanine hydrochloride significantly increased the proportion of Cap^+^ cells (two sample *p*<0.001). Given that Cap^+^ levels are decreased by uracil (a pyrimidine) and increased by guanine (a purine), the level of 1B^4^ capsule switching is dependent on the intracellular ratio of pyrimidine and purine pools. While this dependency may reflect direct sensing of deviations from a specified pyrimidine-purine pool ratio, it could also reflect a complex inter-regulation between the biosynthetic pathways.

In an attempt to distinguish between these possibilities, the above experiment was also performed with 1A^4^ and the *pyrH* switcher, Re1_4; each of these strains was grown in the presence and absence of 1.5 mM guanine hydrochloride. If the pyrimdine:purine ratio is causing capsule switching, one would expect to also see an increase in capsulation in these two genotypes (because the pyrimidine:purine ratio is biased towards purines – a similar effect to knocking down pyrimidine intermediates). However, the addition of 1.5 mM guanine hydrochloride had no significant effect on either Re1_4 or 1A^4^ (two sample *p*>0.1).

The above results indicate that the effect of guanine hydrochloride observed in 1B^4^ results from co-regulation of the pyrimidine and purine biosynthetic pathways, rather than purines directly influencing the switch decision. For example, the addition of purines may negatively regulate some early genes in the pyrimidine biosynthetic pathway, causing further pyrimidine reduction in 1B^4^. An indirect role for pyrimidine:purine ratios in 1B^4^ capsule bistability is further highlighted by the alteration of capsulation levels in several transposon mutants with insertions in nucleotide pathways (S1 Table). These include an insertion in the pyrimidine biosynthetic gene *dcd* (*pflu1304*, encoding deoxycytidine triphosphate deaminase EC 3.5.4.13; see Fig. 2) and three insertions upstream of the purine biosynthetic gene *purU* (*pflu4938*, encoding formyltetrahydrofolate deformylase EC 3.5.1.10). A further seven insertions were obtained in the methionine biosynthetic gene *sahA* (*pflu5720*, encoding adenosylhomocysteinase EC 3.3.1.1): methionine and purine biosynthesis are closely linked, and changes in levels of homocysteine (the SahA product) have been shown to cause defects in yeast purine biosynthesis [2].

**References**

**References**

1. O'Donovan GA, Neuhard J (1970) Pyrimidine metabolism in microorganisms. Bacteriol Rev 34: 278-343.

2. Santhanagopalan V, Coker C, Radulovic S (2006) Characterization of RP 333, a gene encoding CapD of *Rickettsia prowazekii* with UDP-glucose 4-epimerase activity. Gene 369: 119-125.
